# *Myotis fimbriatus* Virome, a Window to Virus Diversity and Evolution in the Genus *Myotis*

**DOI:** 10.3390/v14091899

**Published:** 2022-08-27

**Authors:** Alix Armero, Ruiya Li, Kathrina Mae Bienes, Xing Chen, Jihao Li, Shiman Xu, Yanhua Chen, Alice C. Hughes, Nicolas Berthet, Gary Wong

**Affiliations:** 1 Unit of Discovery and Molecular Characterization of Pathogens, Centre for Microbes, Development, and Health, CAS Key Laboratory of Molecular Virology and Immunology, Institut Pasteur of Shanghai, Chinese Academy of Sciences, Shanghai 200031, China; 2 Viral Hemorrhagic Fevers Research Unit, CAS Key Laboratory of Molecular Virology and Immunology, Institut Pasteur of Shanghai, Chinese Academy of Sciences, Shanghai 200031, China; 3 University of Chinese Academy of Sciences, Beijing 100049, China; 4 Xishuangbanna Tropical Botanical Garden, Chinese Academy of Sciences, Jinghong 666303, China; 5 Cellule d’Intervention Biologique d’Urgence, Unité Environnement et Risque Infectieux, Institut Pasteur, 75015 Paris, France

**Keywords:** virome, *Myotis*, alphacoronavirus, co-specificity

## Abstract

Significant efforts have been made to characterize viral diversity in bats from China. Many of these studies were prospective and focused mainly on *Rhinolophus* bats that could be related to zoonotic events. However, other species of bats that are part of ecosystems identified as virus diversity hotspots have not been studied in-depth. We analyzed the virome of a group of *Myotis fimbriatus* bats collected from the Yunnan Province during 2020. The virome of *M. fimbriatus* revealed the presence of families of pathogenic viruses such as *Coronavirus*, *Astrovirus*, *Mastadenovirus*, and *Picornavirus*, among others. The viral sequences identified in *M. fimbriatus* were characterized by significant divergence from other known viral sequences of bat origin. Complex phylogenetic landscapes implying a tendency of co-specificity and relationships with viruses from other mammals characterize these groups. The most prevalent and abundant virus in *M. fimbriatus* individuals was an alphacoronavirus. The genome of this virus shows evidence of recombination and is likely the product of ancestral host-switch. The close phylogenetic and ecological relationship of some species of the *Myotis* genus in China may have played an important role in the emergence of this alphacoronavirus.

## 1. Introduction

Some of the major epidemic outbreaks related to viruses from the *Coronaviridae*, *Paramyxoviridae*, and *Filoviridae* families were associated with zoonotic events. Phylogenetic and epidemiological evidence suggests that bat viruses may be involved in these interspecies jumps [1,2,3,4,5,6,7,8]. Indeed, bats have shown one of the largest viral diversities among mammals, and perhaps the most relevant in terms of zoonotic viruses [9]. This extraordinary viral diversity is explained by the different biological and ecological characteristics of these organisms. The immune system of bats allows the coexistence of replicative viral populations without the manifestation of clinical symptoms [10,11]. This system appears to be based on a balance between host defense and tolerance to disease [4,12,13,14]. Bats are distributed globally and are the second most diverse group among mammals [15,16]. These mammals roost in foliage, rock crevices and caves, and hollow trees, as well as human-made structures such as barns, houses, and bridges [17]. Moreover, bats exhibit behaviors that may promote viral diversity and zoonotic spillover events. Diverse bat assemblages could be found in caves, where many species will commonly roost together [18]. In these assemblages, infected individuals could shed the virus into the environment, contaminating other individuals [19]. However, perhaps the most important factor that has triggered the emergence of zoonotic viruses is the increasing pressure exerted by human activities on the habitat of bats. Increasing urbanization, deforestation, habitat degradation, roost destruction, and the economic and cultural activities of humans increase the chances of contact with bat viral diversity, as well as potentially increasing individual viral loads [20].

Yunnan Province is an epicenter for biodiversity and endemism in the southwest of China, hosting more than half of China’s species, and is acknowledged as a global biodiversity hotspot [21]. This province is part of a biogeographical region, called the Greater Mekong Subregion, that extends beyond its borders with Vietnam, Lao PDR, Thailand, Cambodia, and Myanmar [22,23]. Multiple studies have explored the viral diversity of bats in Yunnan [24,25,26,27,28,29]. In particular for coronaviruses, sequences similar to SARS-CoV were identified in *Rhinolophus* bats from this region [30,31]. These studies indicate that Yunnan is a potential hotspot for coronavirus diversification in China [32]. Initially, the diversity of coronaviruses and other viral taxa was studied by PCR amplification of conserved genes [2,33,34,35,36]. However, virome studies based on metagenomic and metatranscriptomic approaches have gradually increased in recent times [24,25,26]. Although these studies have made important efforts to cover the diversity of bats, some groups have been prioritized, as is the case of *Rhinolophus*. As such, the viromes and potential pathogens circulating in other relatively neglected bat species from this region remain poorly characterized.

*Myotis fimbriatus* is a species of the genus *Myotis* and the *Vespertilionidae* family. This genus has over 120 species distributed on all continents [16,37,38]. The members of this genus present primitive morphological characteristics and little specialization in functions such as echolocation [39]. *M. fimbriatius* is endemic to China, with a wide geographical distribution in this country [40]. This bat is an insectivorous species that lives in large colonies in caves and abandoned tunnels; *M. fimbriatus* may co-roost with other bat species in these spaces [37,41].

Here, we analyzed the virome of twenty *M. fimbriatus* individuals captured from the same cave in Yunnan Province during 2020. A previous study identified astroviruses, coronaviruses, adenoviruses, and circoviruses in samples of *M. fimbriatus* by PCR amplification [42]. In our study, the viromes were obtained with a targeted molecular approach and next-generation sequencing. The virome of *M. fimbriatus* revealed the presence, prevalence, and abundance of emerging virus families. We also assembled the genome of an alphacoronavirus that was the most abundant and prevalent virus in the individuals. The evolution of this sequence appears to be closely related to the ecology and evolution of some *Myotis* species present in Yunnan Province.

## 2. Materials and Methods

### 2.1. Samples and Sequencing

Bats were sampled using four bank harptraps and mistnets, which were set between 5 pm and 11 pm. Samples were collected on 30 September 2020. The sampling site was a cave in Shilin County, Yunnan Province (Figure S1). Body measurements (Forearm, Tail, Hindfoot, Tibia, Tail, Headbody) were taken using a Mitutoyo Absolute Series-500 calliper with an accuracy of 0.01 mm (Mitutoyo Corporation, Kawasaki, Japan), and bodymass was measured with a Pesola Spring Scale (Pesola^®^ Präzisionswaagen AG compamy, Schindellegi, Switzerland). Tissue samples were obtained from wing membranes of bats collected using a 3 mm biopsy punch. Bats were released after tissue samples, measurements, photographs, and echolocation calls were recorded. Wing tissue samples were stored in vials of 99.7% alcohol. Bat species identification was performed on the basis of species physiology and confirmed using CO1 sequenced in the Southwest Barcoding centre from referenced earlier samples at the same sites (see Chornelia et al. [43] for full methods).

Rectal swabs from twenty *M. fimbriatus* individuals were obtained for metagenomic analysis (Table S1). The rectal samples were later stored in RNA (Thermo Fisher Scientific, Waltham, MA, USA) and transported in dry ice. In the laboratory, the samples were stored at −80 °C. The samples were taken from −80 °C, thawed at 4 °C, vortexed for 3–5 min, and centrifuged at 17,000× *g* at 4 °C for 3 min. The supernatant was collected by pipette and transferred into a 0.45 µM filter microtube (Corning), and then centrifuged at 15,000× *g* at 4 °C for 1 min. The flow-through was used for nucleic acid purification. The nucleic acids of samples were extracted using the GeneJet Viral DNA and RNA Purification Kit (Thermo Fisher Scientific). The extraction was stored at −80 °C for later use.

**Library preparation and target enrichment**. From the RNA extracted from each stool sample collected from the 20 bat individuals belonging to *M. fimbriatus*, a cDNA library creation protocol was performed using the Twist library preparation kit including a targeted enrichment step. In brief, RNA was first converted to cDNA using random hexamers and ProtoScript II First Strand cDNA Synthesis Kit, both from New England Biolabs (NEB). The ssDNA was then converted to dsDNA using NEBNext Ultra II Non-Directional RNA Second Strand Synthesis Kit, also from NEB. Subsequently, DNA fragmentation, end repair, and dA-tailing were carried out, followed by ligation with Twist Universal Adapters. Libraries compatible with Illumina TruSeq were then amplified by PCR using Twist UDI Primers. The targeted enrichment step was performed using the Twist target enrichment standard hybridization protocol with some modifications. This protocol involves the enrichment of genomic DNA libraries with a 16 h hybridization in a two-day workflow using the Pan-Viral Panel from Twist Bioscience consisting of 600,000 DNA probes, allowing target enrichment of over 1000 viral human pathogens. However, unlike the standard protocol, a second set of custom panels of probes from Agilent Technologies that targeted 67 CoV whole genomes was added in the hybridization capture step (1 μL of each probe set instead of 2 μL of Twist Pan-Viral Panel of probes). Then, targets were captured using beads, and post-capture PCR amplification was performed to enrich the captured targets. Following enrichment, libraries were sequenced on the Illumina NovaSeq platform with 150 bp paired-end reads (Novogen). Raw reads are openly available in the NCBI, Bioproject reference number PRJNA865499.

### 2.2. Bioinformatics Analysis

**Taxonomic classification**. Trimmomatic tool (v. 0.39) trimmed low-quality regions and adapters from the reads [44]. The clean reads were aligned with Bowtie2 (v. 2.4.5), [45] to the genomes and transcriptomes of eight different bat species obtained from NCBI [46]. The reads that did not align against the bat sequences were realigned with Bowtie2 against the human genome assembly GRCh38 [47]. MEGAHIT software (v. 1.2.9), [48] performed de novo assembly on reads that did not align with the human sequences. Only sequences with a minimum length of 100 nucleotides were retained. The CD-HIT-EST software (v. 4.8.1) clustered de novo contigs according to 99% nucleotide identity and 100% coverage for the shortest reads [49]. The longest sequence of each of these clusters was retained for further analysis. The clean reads were aligned to de novo contigs with Bowtie2. BBmap tool (v. 38.96) estimated the reads per kilobase per million mapped reads (RPKM) in the alignment and the average fold coverage [50]. The contigs supported by the reads were aligned with BLASTN (v. 2.12.0), (e-value < 1 × 10^−10^) against the reference viral database (RVDB) version 22 [51]. The best hit for each of the contigs was identified using the e-value, identity, and alignment coverage as selection criteria, in this order of importance. The taxonomic classifications of viral sequences with vertebrates and/or invertebrates hosts were manually checked. Contigs aligning to the same genomic sequence were regrouped and aligned with LASTZ (v. 1.04.15), [52] to the respective genomic sequences to determine coverage.

Here, 0.87 Giga clean reads were obtained after trimming reads and low-quality regions; 81% (0.70 Giga) of clean reads aligned to bat sequences, while only 0.04% (374,063) of reads aligned to the human genome. From the remaining reads, 3450 viral contigs were assembled; 2,181,815 reads aligned against these contigs, representing 0.25% of clean reads (Table S2). The median of the average fold coverage per contig was 49.61, while the minimum and maximum values were 1.99 and 79,046.20, respectively. Table S3 presents the results of the BLASTN alignments for 3450 viral contigs and some of the main statistics of the alignment of the reads to the contigs.

**Phylogenetic analysis of virus families with vertebrate hosts**. For some of the virus families with vertebrate hosts, phylogenies were reconstructed. Initially, multiple alignments were obtained, including representative sequences and the contigs with the MAFFT tool (v. 7.505), [53]. Multiple sequence alignments were inspected and cured with MEGAX (v. 10.1.7), [54]. The best sequence evolution model was identified with jmodeltest (v. 2.1.10), [55] and ProtTest3 (v. 3.4.2), [56] in the nucleotide and amino acid alignments, respectively. Phylogenetic trees were reconstructed with MrBayes software (v. 3.2.7a), [57]. The number of generations varied between 1 and 5 million, sampled every 500 generations, with 10% burnin removed. Convergence of the posterior probabilities was assessed by checking the standard deviation of split frequencies and the resulting PSRF statistics. The analyses were stopped when the average standard deviation was less than 0.01. The resulting trees were represented in R (v. 4.1.2), [58], with the packages ggnewscale (v. 0.4.7), [59], ggtree (v. 3.3.6), [60], and treeio (v. 1.20.2), [61]. Phylogenetic analysis was performed with MrBayes for the following families (gene and alignment length are indicated in parentheses): pedacovirus (ORF1b: 291 a.a, spike: 141 a.a), astrovirus (ORF1b: 260 a.a, capsid: 564 a.a), mastadenovirus (pVI: 184 a.a), picornavirus (peptidase C3: 84 a.a), and poxvirus (DNA-dependent RNA 360 polymerase 132 kDa subunit gene: 242 bp).

**BtMf-Yunnan2020 genome assembly**. The same library for individual 14 that had been previously sequenced was again sequenced with a greater depth (~145 millions of paired-end reads) on the Illumina NovaSeq 6000 sequencer (Novogen, Shanghai, China). The reads were trimmed with Trimmomatic and assembled into the contigs with MEGAHIT. Clean reads and contigs were then assembled into scaffolds with metaSPAdes [62]. The reads were aligned to the scaffolds with Bowtie2 to verify assembly. The similarity between myotacovirus genomes was plotted with the seqcombo packages in R.

**Phylogenetic analysis of BtMf-Yunnan2020 genome**. Representative alphacoronaviruses genomes were obtained from NCBI [47]. A multiple sequence alignment was obtained with MAFFT. TrimAl software (v. 1.2), [63] removed regions of low quality in multiple sequence alignment. The jModelTest identified the best model for nucleotide substitution. MrBayes software inferred phylogenetic trees from multiple alignments of the whole genome, spike, and subunit 1 of the alphacoronaviruses. PhyML (v. 3.3.2), [64] inferred the phylogenetic tree from an alignment of a 5 kb region of alphacoronaviruses ORF1B gene. Two Middle East respiratory syndrome (MERS) genomic sequences from the *Betacoronavirus* genus were used as an outgroup in the phylogenetic analysis.

## 3. Results

### 3.1. Virome Profile

Viruses with vertebrate hosts were represented by eight families, eleven genera, and unclassified sequences; these sequences were observed in thirteen samples. This group was the most abundant, representing 85.90% (1,874,276) of the reads that aligned to the contigs. The bacteriophages were the second-most abundant group; these viruses were classified into nine families and sixty-two genera. Bacteriophages represented 13.82% (301,631) of viral reads and were observed in all samples. Viruses associated with vertebrate and invertebrate hosts were represented by *Peribunyaviridae* and *Nodaviridae* families; the genus *Orthobunyavirus* represented the first of these families, while unclassified sequences represented the nodaviruses; these viruses were detected in three samples and only 0.24% (5331) of the reads aligned to these sequences. The *Dicistroviridae* family represented the viruses with invertebrate hosts; the *Cripavirus* genus and unclassified sequences represented this family in five samples; 0.02% (445) of reads were associated with this group. The *Virgaviridae* family and the *Tobamovirus* genus represented plant viruses; these viruses were observed in three samples and 0.01% (132) of the reads aligned to this group (Table S4).

### 3.2. Viruses with Vertebrate Hosts

**Figure 1:**
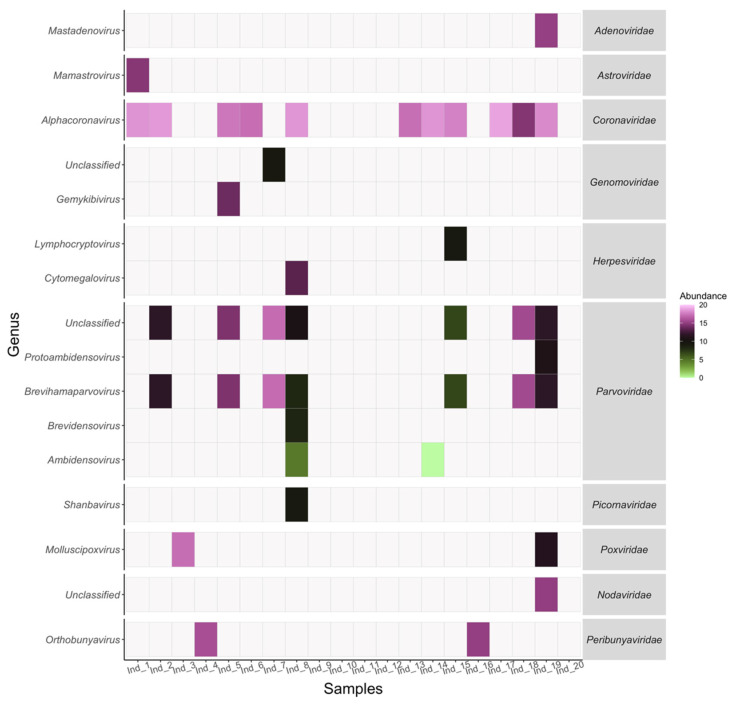
Virome presence and abundance. The abundance within samples is presented for genera and families in viruses with vertebrate hosts. The abundance is expressed by log2 of the reads per kilobase per million mapped reads (RPKM).

Several viral families related to pathogenesis and zoonotic events in humans and livestock were identified in *M. fimbriatus* (Figure 1).

#### 3.2.1. Pedacovirus

**Figure 2:**
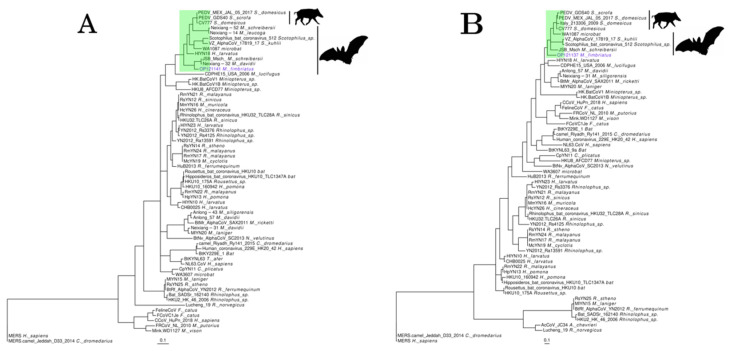
Pedacovirus. Phylogenetic trees of representative alphacoronavirus sequences were obtained for (**A**) a 291 a.a region of the ORF1b protein and (**B**) a 141 a.a region of the spike. The trees were inferred under a general matrix model (LG), with gamma-distributed rate variation among sites (G) and the proportion of invariable sites (I). *Pedacovirus* subgenus is highlighted in green.

*Pedacovirus* is a member of the *Alphacoronavirus* genus within the *Coronaviridae* family. Sequences belonging to this subgenus were observed in six samples. However, this subgenus was significantly represented only in individual 19. A group of four contigs (OP121136, 39, 40, 41; NCBI accessions) from this sample covered 10% (1419 bp) of Anlong Ms bat coronavirus (KF294382.1). Anlong Ms bat coronavirus is an ORF1ab gene partial fragment identified in *Myotis davidii* (*M. davidii*) bats [65] (Table S5). Another group of three contigs (OP121137, 38, 42) covered 5% (1431 bp) of the genome of Jingmen Miniopterus schreibersii alphacoronavirus 2 (MZ328300.1). This genomic sequence was obtained from the *Miniopterus schreibersii* (*M. schreibersii*) bats [66] (Table S5). *M. fimbriatus* contigs aligned against the spike, ORF8, and membrane genes of Jingmen Miniopterus schreibersii alphacoronavirus 2. In both cases, nucleotide identities were low (81–86%). Phylogenetic trees of partial regions of ORF1b and spike proteins confirmed the phylogenetic relationship of the *M. fimbriatus* contig to the pedacovirus clade (Figure 2). This is the clade of the pathogenic porcine epidemic diarrhea virus (PEDV) responsible for a devastating enteric disease in feeder pigs [67]. In the ORF1b phylogenetic tree, the *M. fimbriatus* contig was found on a solitary branch sister to a sub-cluster composed of Anlong Ms bat coronavirus and Jingmen Miniopterus schreibersii alphacoronavirus 2. In the analyzed region, the amino acid identity between *M. fimbriatus* and these sequences was ~91%, while this identity was 78% and 82% in relation to Scotophilus bat coronavirus and PEDV, respectively. In the spike tree, the divergence between the pedacovirus of *M. fimbriatus* and the sequences of this subgenus was significant. The sequence of *M. fimbriatus* had an identity of 84% in relation to Jingmen Miniopterus schreibersii alphacoronavirus 2, between 76 and 78% for the other bat viruses, and between 77 and 81% in relation to the PEDV sequences.

#### 3.2.2. Astrovirus

**Figure 3:**
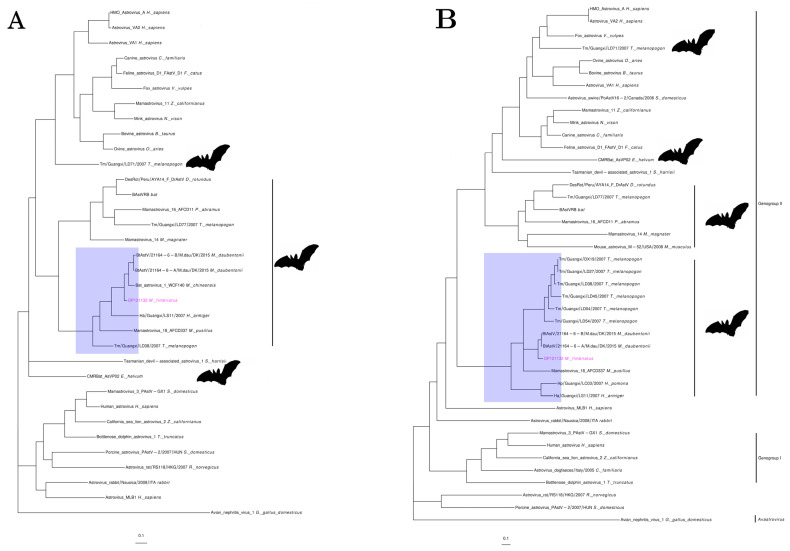
Astrovirus. Phylogenetic trees of representative sequences of astrovirus in (**A**) a 260 a.a region of ORF1b and (**B**) a 564 a.a region of the capsid protein. Both trees were inferred under an LG + I + G model.

Individual 1 presented sequences (OP121130-3) that covered 74% (4812 bp) of two Bat astrovirus genomes (MZ218053.1, MZ218054.1). These Bat astrovirus are part of the *Mamastrovirus* genus of the *Astroviridae* family and were assembled from metagenomic samples of *Myotis daubentoniid* (*M. daubentoniid*) bats [68] (Table S5). The *M. fimbriatus* sequences had significant divergencies to the three ORFs of these mamastroviruses. Nucleotide alignments were not obtained for ORF1a and the first 327 nucleotides of the capsid genes. For the remaining region of the capsid, the identity was low (79%). In ORF1b, the identity increased towards the end of this gene (83–91%). Amino acid identity was low for ORF1a and capsid (75% and 81%, respectively) and more corserved for ORF1b (89–98%). The phylogenetic tree of the capsid retrieved the two genogroups of mamastroviruses recognized and used in the taxonomic classification within this genus (Figure 3) [69]. Two clusters with only bat viruses were found within genogroup II together with the “human mink ovine astroviruses” clade. The sequences of *M. fimbriatus* belonged to a cluster populated by bat viruses. This cluster included sequences from *Emballonuridae*, *Hipposideridae*, and *Miniopteridae* bat families sampled in China and Hong Kong [69,70,71]. Within this cluster, sequences belonging to the same bat species/genus were grouped together. In the ORF1b phylogenetic tree, the two bat clusters of genogroup II formed a large clade. In this tree, the *M. fimbriatus* viral sequence was related to the *M. daubentoniid* sequences and Bat astrovirus 1 (EU847146.1); this bat astrovirus was identified in *Myotis chinensis* (*M. chinensis*) bats [72] (Table S5). The amino acid identity between the *M. fimbriatus* contig and the *M. daubentoniid* sequences in the capsid bat cluster was low (88%); in comparison, the identity between *M. daubentoniid* sequences was high (99%). In the ORF1b bat cluster, the identity of *M. fimbriatus* contig was important in relation to the sequences of *M. daubentoniid* and *M. chinensis* (92%), while the identity between *M. daubentoniid* sequences was high (98%).

#### 3.2.3. Mastadenovirus

**Figure 4:**
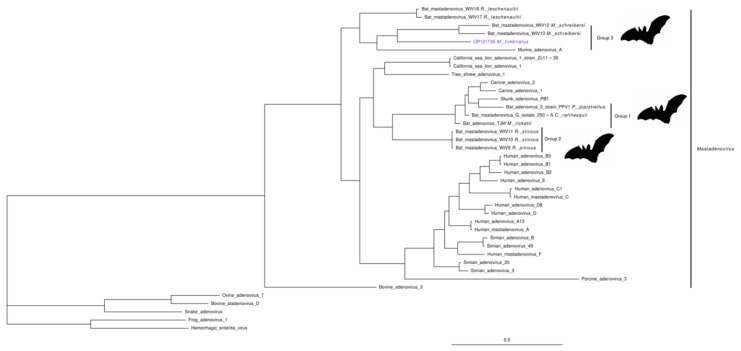
Mastadenovirus. Phylogenetic tree of a 184 a.a region of the pVI protein of representative sequences of the *Mastadenovirus* genus. Bayesian inference was based on an LG + I + G model. The three groups of bat mamastroviruses are indicated.

Two contigs from individual 19 were related to hexon and pVI genes of bat mastadenoviruses. *Mastadenovirus* is a genus of the family *Adenoviridae*. A *M. fimbriatus* contig (OP121134, 630 bp) aligned with an identity of 95% against Bat adenovirus N78-28/Germany/2008 (HM368167.1); this is a partial sequence of the hexon gene associated with *Myotis Myotis* (*M. Myotis*) bats [73], (Table S5). The second contig (OP121135, 765 bp) did not have a significant nucleotide alignment; the amino acid sequence of this contig aligned with low identity (60%) to the pVI polypeptide of Rousettus aegyptiacus adenovirus (AXE75632.1) [74] (Table S5). In the pVI phylogenetic tree, the *M. fimbriatus* contig clustered with group three of bat mastadenovirus (Figure 4). Three different groups of bat mastadenoviruses have been identified to date [75]; group three is composed of sequences identified in *M. schreibersii* bats (BtAdv WIV12/WIV13) and *Rousettus leschenaultii* bats (BtAdv WIV17/WIV18) (Figure 4). The low amino acid identity (52–57%) and the long branch linking the *M. fimbriatus* contig to the other sequences in group three suggest low conservation.

#### 3.2.4. Picornaviruses

**Figure 5:**
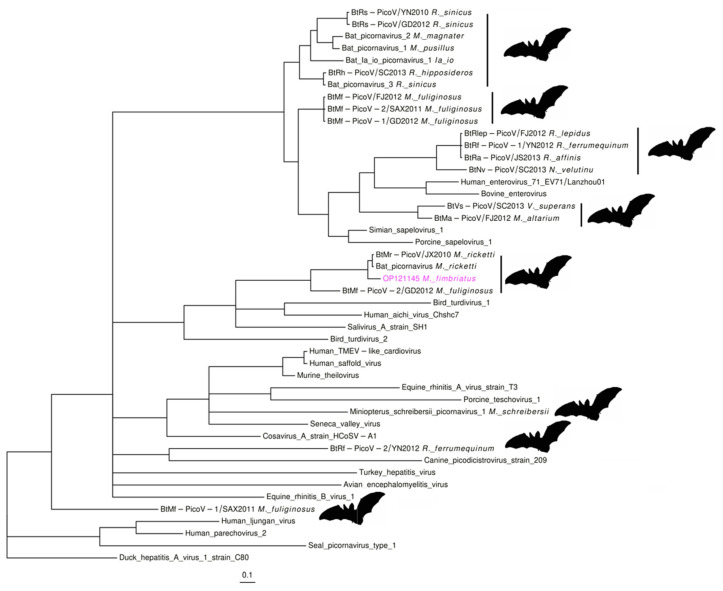
Picornavirus. Phylogenetic tree of an 84 a.a region of the peptidase C3 protein. The tree was derived under an LG + I + G model.

In the *Picornaviridae* family, three contigs from individual 8 (OP121143-5; 267–381 bp) covered 11.5% (~870 bp) of Bat picornavirus (NC_043071.1). Bat picornavirus is a partial genome sequence from *Myotis ricketti* (*M. ricketti*) bats [34] (Table S5). The identity between the viral sequences of these bats suggests some degree of divergence, mainly between the nucleotide sequences (85–91%: nucleotide; 93–97%: amino acid). Two of the contigs aligned to unannotated regions; the remaining contig aligned to the gene coding the peptidase C3. Phylogenetic analysis in a region of this gene placed the *M. fimbriatus* contig with bat picornaviruses from *Myotis* and *Miniopterus* (Figure 5). This group corresponds to clade four of bat picornaviruses identified in a previous study [34]. The amino acid identity was important between the sequences of *M. ricketii* and *M. fimbriatus* (92–98%) and low between these contigs and the *Miniopterus fuliginosus* sequence (51–74%). This group of bat picornaviruses seems to be phylogenetically close to *Kobuvirus* and *Salivirus* sequences (Figure 5). The amino acid identities between the group of bat picornaviruses and the *Kobuvirus* and *Salivirus* sequences were low (<60%).

#### 3.2.5. Poxviridae

**Figure 6:**
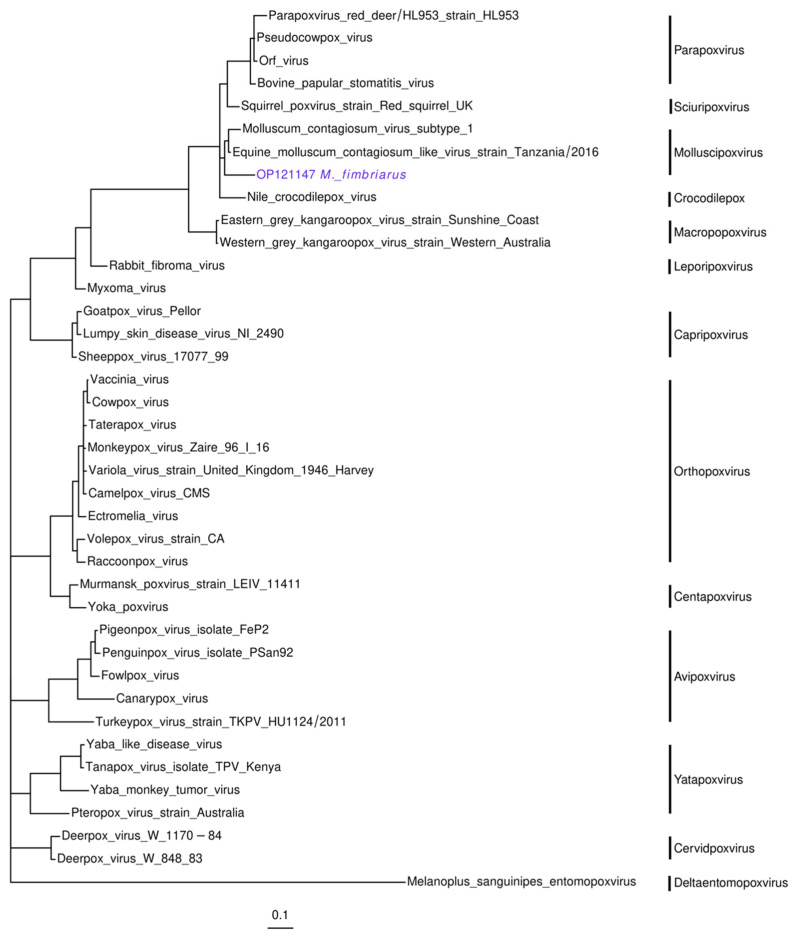
Poxvirus. Phylogenetic tree of the nucleotide alignment of a 242 bp region of the DNA-dependent RNA polymerase 132 kDa subunit gene. The inference of the tree was based on a general time-reversible (GTR) model, with gamma-distributed rate variation among sites and the proportion of invariable sites.

Individuals 3 and 19 presented three contigs (OP121146-8; 147–243 bp) associated with the Equine molluscum contagiosum-like virus (MN339351.1). Equine molluscum contagiosum-like virus belongs to the *Molluscipoxvirus* genus in the *Poxviridae* family. This genome (167 Kb) was recently sequenced from biopsies of horses with papular dermatitis [76]. The contigs aligned against the genes coding the 132 kDa and 147 kDa subunits of the DNA-dependent RNA polymerase. The identity between some of these sequences was significant (89–94%: nucleotide; 94–100%: amino acid). The phylogenetic analysis placed one of these contigs in the molluscipoxviruses clade. In this cluster, the Molluscom contagiosum virus and the Equine molluscom contagiosum-like virus formed a subclade, while the *M. fimbriatus* sequence diverged in a sister branch (Figure 6). In the aligned region, the amino acid identity between the Molluscom contagiosum virus and the Equine molluscom contagiosum-like virus was relatively significant (95%), while the identity between these sequences and the contig of *M. fimbriatus*. 

#### 3.2.6. Herpesvirus and Genomovirus

The virome of *M. fimbriatus* also presented some evidence of the presence of the families *Herpesviridae* and *Genomoviridae* (Table 1). The *Lymphocryptovirus* and *Cytomegalovirus* genera were represented by single contigs with high identities (>97%) to the genes coding BORF2 and US26 proteins, respectively. In the family *Genomoviridae*, a contig had 100% identity to a small region of the *Rep* gene of *Molossus molossus*-associated gemykibivirus 5 (*Molossus molossus* bats).

### 3.3. Viruses with Vertebrate and Invertebrate Hosts

**Densovirinae.** This *Parvoviridae* subfamily was represented mainly by viruses infecting mosquitoes of the *Culex* and *Aedes* genera. These contigs had high identities to the target sequences and were observed in eight individuals (Figure 1) (Table 1). Three of the contigs had unequivocal alignment to sequences obtained from the sugarcane borer (*Diatraea saccharalis*), the house mosquito (*Culex pipiens*), and the Eurasian tree sparrow (*Passer montanus*). The first two contigs aligned against the non-structural protein 1, while the last one aligned against the VP1 structural protein. The remaining nine contigs were categorized as unclassified densovirinae and had multiple best hits. These contigs aligned with the same identity against densoviruses from *Culex* and *Aedes* mosquitoes. According to the best hits, these contigs were divided into three groups; for each of these groups, two targets are presented in Table 1 representing densoviruses of both mosquito genera. The contigs of the three groups aligned against the gene coding the non-structural protein 1.

**Peribunyaviridae and Nodaviridae.** Some evidence was found for the genus *Orthobunyavirus* of the family *Peribunyaviridae*. The reads aligned with a significant identity (>96%) to the glycopolyprotein RNA-dependent RNA-polymerase gene of Oropouche virus and Orthobunyavirus FSL2923. Two contigs represented the *Nodaviridae* family. One of these contigs (681 bp) aligned with low identity (88%) to a bat nodavirus from the *Eptesicus serotinus*. Similarly, the second contig (490 bp) aligned with low identity (87%) against the putative polymerase of Xinjiang sediment noda-like virus 1 (Table 1).

### 3.4. Bacteriophages

Bacteriophages were the most abundant group of viruses with non-vertebrate hosts in the *M. fimbriatus* virome (301,631 reads). *Myoviridae* and *Autographiviridae* families were the most abundant and diverse among the bacteriophages; these families were observed in all samples. The sequences of the *Myoviridae* family were classified into twenty-one genera; sequences of unknown genus were also identified in this family. In total, 73.25% (220,945) of the reads that were aligned to bacteriophage sequences belonged to the *Myoviridae* family. The second-most abundant family among the bacteriophages was *Autographiviridae.* This family presented ten genera and represented 22.41% (67,608) of the reads that aligned against the bacteriophage sequences (Table S4).

### 3.5. Genomic Sequences of a M. fimbriatus Myotacovirus

**Figure 7:**
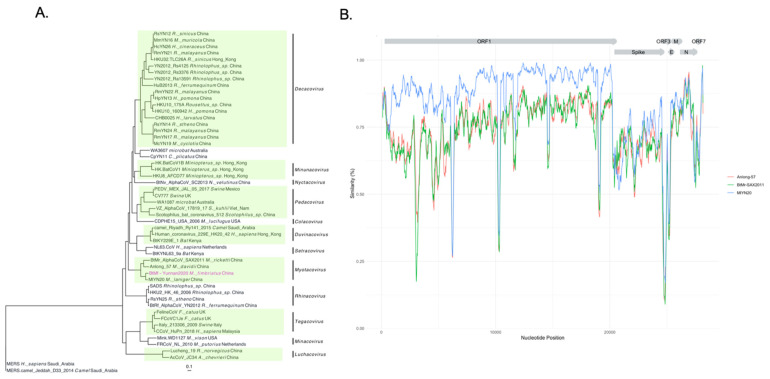
Phylogenetics and similarity of the BtMf-Yunnan 2020 genome. (**A**) A phylogenetic tree was inferred from the alignment of the nucleotide sequences of the alphacoronavirus genomes (23,544 bp). The inference was based on a GTR + I + G model. Alphacoronavirus subgenera are indicated. (**B**) Similarity profile of myotacovirus sequences to the BtMf-Yunnan2021 genome.

The most abundant and prevalent virus in the *M. fimbriatus* virome was an alphacoronavirus. This virus was identified in six samples and represented 84% (1,825,267) of viral reads (Figure 1). We assembled the genome of this alphacoronavirus by deep sequencing of one of the samples. This sequence was called BtMf-Yunnan2020 (OP279992) and has a length of 27,205 bp. Phylogenetic analysis with representative sequences of the *Alphacoronavirus* genus showed that this genome belongs to the subgenus *Myotacovirus* (Figure 7A). This clade is currently composed for BtMr-SAX2011 (*Myotis ricketii*), Anlong-57 (*Myotis davidii*), and MlYN20 (*Myotis laniger*). In the phylogenetic tree, BtMf-Yunnan2020 and MlYN20 clustered together, a close relationship confirmed by significant structural and sequence similarity. BtMf-Yunnan2020 and MlYN20 shared the structural organization of the genome 5′-ORF1ab-Spike-ORF3-E-M-N-ORF7-3′ (Figure 7B). However, BtMf-Yunnan2020 presented a deletion of ~70% of the ORF3 gene in comparison with MlYN20. The identity in the replicase conserved domains [77] between BtMf-Yunnan2020 and MlYN20 was the highest (>95%) among all pairwise comparisons in myotacoviruses (Figure S1). Although phylogenetically related, myotacoviruses exhibited low amino acid sequence identity. Most of the pairwise comparisons between myotacoviruses proteins had identities <90% (Figure S2). In the spike, only the coupled BtMf-Yunnan2020-MlYN20 and Anlong-57-BtMr-SAX2011 had an identity >75%. The amino acid identity did not exceed 70% for any pairwise comparisons in ORF3 and ORF7.

**Figure 8:**
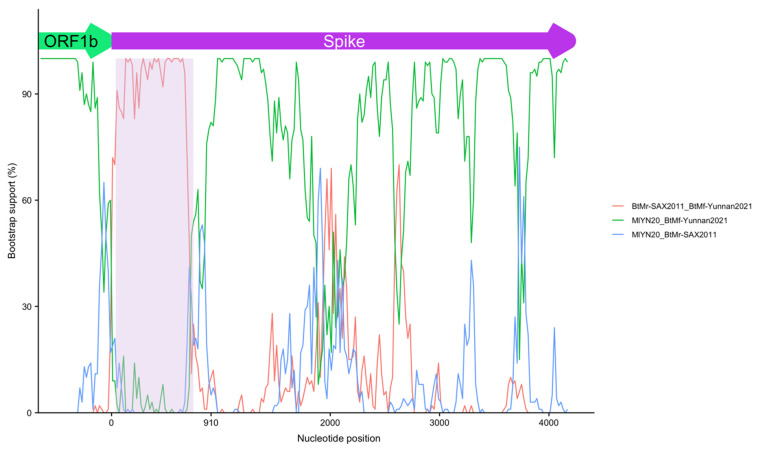
Spike recombination. Bootstrap support for recombination in subunit 1 of the spike protein of BtMf-Yunnan2020. The recombination involves the first 750 nucleotides of the spike (shaded area). The BtMr-SAX2011 spike was identified as the minor parent in this recombination.

The similarity profile showed a change in the initial part of the BtMf-Yunnan2020 spike gene, characterized by a fall in relation to MlYN20 and an increase in relation to BtMr-SAX2011 and Anlong-57 (Figure 7B). This abrupt change in the similarity profile suggests a recombination event. This hypothesis was evaluated with RDP4 software (v. 1.0), [78]. This software identified a recombination region at the 5′ end of the spike gene. The beginning breakpoint was identified 40 nucleotides after the start of this gene, while the ending breakpoint was located at position 750 (Figure 8). The MlYN20 and BtMr-SAX2011 spikes were identified as the major and minor parents, respectively. Despite this recombination, BtMf-Yunnan2020 and MlYN20 clustered together in the phylogenetic trees of spike proteins and subunit 1 (Figure S3).

#### Diversity between Individuals of the BtMf-Yunnan2020 Genome

**Figure 9:**
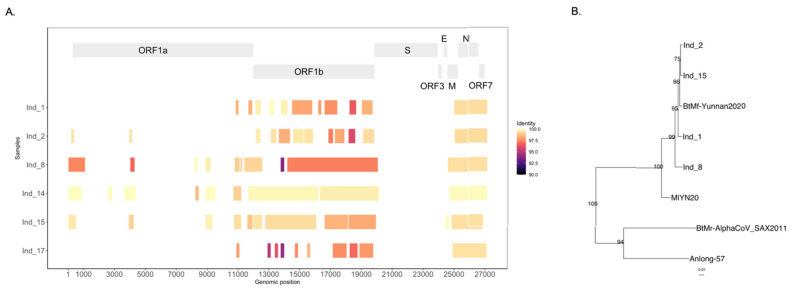
Inter-host diversity of the BtMf-Yunnan2020 genome. (**A**) Identity and breadth of coverage of BtMf-Yunnan2020 sequences in six individuals of *M. fimbriatus*. (**B**) Expansion in the myotacovirus subclade of a phylogenetic tree inferred from a 5 kb region of alphacoronavirus ORF1b; four consensus sequences from individuals were included in this analysis; statistical support is indicated.

The BtMf-Yunnan2020 genome was associated with 58 contigs in six samples. Figure 9A shows the breadth of coverage and nucleotide identity between these contigs and the BtMf-Yunnan2020 genome. The median breadth of coverage of the BtMf-Yunnan2020 genome was 34.1% [21%–51%]; the contigs were distributed asymmetrically, concentrating on ORF1ab, nucleocapsid, and ORF7. The median nucleotide identity between these contigs and the BtMf-Yunnan2020 genome was 99.07% [93.88–100%]; 55% (32) of these contigs had a nucleotide identity >99%, while 70.7% (41) of the contigs had an identity >98%.

Comparatively, the median of nucleotide identity of these contigs to the MlYN20 genome was 94.4% [81.44–97.68%]. The nucleotide differences in relation to the BtMf-Yunnan2020 genome varied between individuals. For example, in the ORF1b gene, the nucleotide identities between individuals 8 and 15 and BtMf-Yunnan2020 were 98.2% and 99.3%, respectively (Figure 9A). A phylogenetic tree was inferred involving an ORF1b region (5 Kb) for four individuals, the BtMf-Yunnan2020 genome, and alphacoronavirus sequences (Figure 9B). This phylogenetic tree confirms with high replicability that the sequences identified in individuals were closely related to the genome BtMf-Yunnan2020 (Figure 9B).

## 4. Discussion

### 4.1. The Virome of an Insectivorous Bat

The virome of *M. fimbriatus* presented groups of viruses that may be related to the diet of this insectivorous bat. *Dicistroviruses* and *densoviruses* were identified in a significant number of individuals. Various studies of insectivorous bats identified these groups of viruses mainly in fecal samples [79,80]. Bacteriophages were present in all individuals and were the second-most abundant group [81,82]. This group was dominated by the *Myoviridae* and *Autographiviridae*, but other families previously related to insectivorous bats were also identified, as is the case of *Siphoviridae*, *Podoviridae*, and *Inoviridae* [35,83,84]. The prevalence and abundance of bacteriophages may reflect their active replication within the bacterial hosts of the bat microbiome. In contrast, dicistroviruses and densoviruses are possibly transported through the digestive system without infecting the cells of the mammalian host. Some other viral taxa also possibly associated with the insectivorous behavior of *M. fimbriatus* were observed. This is the case of orthobunyaviruses, nodaviruses, and genomoviruses. Together, these results reflect the potential that rectal samples offer to characterize different aspects of bat ecology.

### 4.2. Families of Pathogenic Viruses

The viral families with vertebrate hosts identified in *M. fimbriatus* were characterized by significant diversity and a tendency for co-specificity between the virus and the bat host. This co-specificity was represented by the phylogenetic clustering of viruses hosted by bats of the same genus or species. A significant divergence of *M. fimbriatus* sequences from previously known bat sequences was observed. These sequences could represent new viral species in some instances. In these families, clusters composed exclusively of bat viruses had phylogenetic relationships with viruses from other mammals; these relationships involved human, bovine, ovine, canine, feline, porcine, equine, and simian viruses, among others.

#### 4.2.1. Pedacovirus

The family *Coronaviridae* consists of enveloped viruses with positive-sense, single-stranded RNA genomes (27–31 Kb). Within the family *Coronaviridae*, the *Orthocoronavirinae* sub-family has four genera: *Alphacoronavirus*, *Betacoronavirus*, *Gammacoronavirus*, and *Deltacoronavirus* [85]. *Alphacoronavirus* and *Betacoronavirus* genera infect mammals and present significant diversity in bats [86]. Several members of the *Alphacoronavirus* genus infect humans, causing mild upper respiratory diseases; this is the case for NL63 [87] and HCoV-229E [85,88,89]. Alphacoronaviruses also infect domestic animals [90,91] and livestock [67,92,93]. We found evidence of alphacoronaviruses of pedacovirus subgenus in *M. fimbriatus*. The *M. fimbriatus* sequence was associated with Anlong Ms bat coronavirus and Jingmen Miniopterus schreibersii alphacoronavirus 2. Our results suggest that these sequences are more closely related to each other than to other bat pedacoviruses, reinforcing the hypothesis that these sequences could represent new species, as previously suggested for Anlong Ms bat coronavirus [65]. Although the sequences of *M. fimbriatus* presented a relatively low identity in relation to the sequences of PEDV, these sequences reinforce the hypotheses that suggest a host-switch between bats and pigs as the origin of the porcine virus [94,95]. Our findings add to a recent analysis that assembled a new pedacovirus genome hosted by *Myotis chilensis* bats [96].

#### 4.2.2. Astrovirus

*Astroviruses* are non-enveloped spherical viruses, with a small single-stranded positive-sense RNA genome (6–10 Kb). This family has two genera, *Avastrovirus* and *Mamastrovirus*, which have traditionally been associated with birds and mammals, respectively [69]. These viruses are responsible for gastroenteritis, respiratory illness, and encephalitis in their hosts [97]. It was previously proposed that *M. daubentoniid* viral sequences might represent a new species, once these sequences clustered independently from other bat mamastroviruses [69]. Similarly, we suggest that *M. fimbriatus* bat mamastrovirus represents a new species; this proposition is supported by the low amino acid identity (81%) and the solitary branch of this sequence in the capsid phylogenetic tree. Previous studies have suggested a significant co-specificity between bat mamastroviruses and the host [70,98,99]. Our results represent new evidence that supports this tendency of bat mamastroviruses. In the phylogenetic analyses of the capsid and ORF1B, the *M. fimbriatus* mamastrovirus belonged to clusters composed exclusively of bat mamastroviruses. Within this cluster, subclusters were defined by viral sequences obtained from the same bat species. In addition, geographic location appears to be less important than the phylogenetic relationship between hosts in this cluster, as shown by the fact that the *M. fimbriatus* sequences formed a subclade with the *M. daubentonii* sequences from Denmark. Vespertilionid species are common across Eurasia, providing a wide range of possible hosts across the region, but a lack of research highlights major gaps in our knowledge of the group. Taken together, these results support species-specificity for this group of bat mamastroviruses.

#### 4.2.3. Mastadenovirus

The family *Adenoviridae* consists of non-enveloped viruses containing a single linear double-stranded DNA genome (26–48 Kb). This family is composed of five genera: *Mastadenovirus*, *Aviadenovirus*, *Atadenovirus*, *Siadenovirus*, and *Ichtadenovirus*. The *Mastadenovirus* genus contains viruses exclusively infecting mammals [100]. Most mastadenoviruses cause a primary infection that generally results in a mild, transient, and self-limited respiratory or enteric illness [101]. Previously, three clusters of bat sequences were identified in a whole-genome phylogenetic analysis [75]. These bat mastadenoviruses groups have been associated with viruses from other mammals, as is the case of group one, which has a close phylogenetic relationship to canine adenovirus [102]. Our analyses of a region of a pVI protein suggest that *M. fimbriatus* sequences belong to group three of bat mastadenoviruses [75]. This is a cluster that contained viruses from different bat genera; within this cluster, the sequences were grouped according to host, suggesting specificity. The low identity of the sequences of *M. fimbriatus* in relation to the other members of this group and their position in a solitary long branch suggest that this specificity would also be supported for the sequences of *M. fimbriatus*. Besides this specificity, group three has been genetically related to California sea lion, a result that suggests a complex phylogenetic landscape in bat mamastroviruses [75].

#### 4.2.4. Picornavirus

Picornaviruses are enveloped viruses with a small single-stranded RNA genome with positive polarity (7–9 Kb). This family is composed of sixty-three genera [103]. The picornaviruses infect a wide variety of animals, and they are responsible for respiratory, cardiac, hepatic, neurological, mucocutaneous, and systematic diseases [104]. Some evidence exists of potential zoonotic events in this family, involving rodent and porcine picornaviruses infecting humans [105]. We found some potential picornavirus in the *M. fimbriatus* samples. Previously, four groups of bat picornaviruses were identified in different provinces of China and Hong Kong. These clusters of bat picornaviruses are composed of viruses from different bat genera and present phylogenetic associations with viruses from other mammals such as porcine and simian sapelovirus 1 [106,107]. Within these bat picornavirus clusters, the sequences were regrouped according to the bat genus [34]. Our results support these trends. The *M. fimbriatus* picornavirus formed a subclade with *M. ricketii* sequences within clade four. These *Myotis* sequences showed low identity to the bat picornavirus of the genus *Miniopterus*, the remaining member of this clade. Our phylogenetic analysis was concordant with previous studies suggesting that clade four of bat picornaviruses appears to be associated with the genus *Kobuvirus*, unlike the other bat picornaviruses clades associated with the *Sapelovirus* genus [34]. *Kobuvirus* have been identified in humans and in some important livestock species, such as cattle and pigs [108]. These viruses are related to gastroenteritis in humans and probably also cause diarrhea in cattle and swine; kobuvirus is transmitted through physical contact or by consumption of contaminated food or water via a fecal–oral route [109]. Some evidence exists of interspecies jumps in this group, for example, between bats and rabbits [110].

#### 4.2.5. Poxvirus

The viruses from the *Poxviridae* family are brick or ovoid-shaped with a double-stranded DNA genome (130–350 Kb). This family is divided into the *Chordopoxvirinae* and *Entomopoxvirinae* subfamilies, which infect vertebrates and insects, respectively [111]. *Poxviruses* can be host-specific or have a wide range. These viruses are responsible for serious infections in livestock and humans, and *Chordopoxvirinae* species often emerge as zoonoses in humans [111]. We identified sequences that could be related to the *Molluscipoxvirus*, the genus of molluscom contagiosum-like virus. Humans are considered to be the only host of this virus, which causes a benign disease that manifests as small umbilicated papules [112,113,114]. However, there is evidence of molluscom contagiosum-like viruses that cause similar diseases in other mammalian species such as donkeys, bats, and kangaroos [115,116,117]. The presence of poxviruses in *M. fimbriatus* should be taken with caution given our limited evidence. However, previously, the presence of a molluscom contagious-like virus was demonstrated in a bat metagenome [117] and our sequences presented a significant divergence; together, these results promote the investigation of molluscipoxvirus in bats.

### 4.3. BtMf-Yunnan2020 Alphacoronavirus

#### 4.3.1. Genomic Sequence and Diversity

In this study, we assembled the novel BtMf-Yunnan2020 genome, contributing to the knowledge of the diversity of alphacoronaviruses in the genus *Myotis*. Phylogenetic analysis classified this genome as a myotacovirus and, according to the species demarcation criteria proposed by the international committee taxonomy viruses (ICTV), the BtMf-Yunnan2020 genome and MlYN20 would represent the same species. Furthermore, these sequences share the same structural organization. However, several characteristics of the BtMf-Yunnan2020 genome make it unique. The BtMf-Yunnan2020 genome showed a deletion in a large part of the ORF3 gene. In PEDV, the ORF3 gene codes a viroporin protein [118], which likely promotes an adequate environment for cellular propagation [119]. PEDV strains adapted to cell cultures presenting deletions in the ORF3 gene produced less severe infections in piglets when compared with the PEDV wild-type genotype. This result suggests an association of ORF3 with PEDV virulence [120]. Whether the ORF3 of *M. fimbriatus* plays a similar role is an area for future investigation. The BtMf-Yunnan2020 genome had a divergent spike protein. The divergence was even more important for subunit 1, in which a recombination spanning the first 236 nucleotides was identified. Subunit 1 is responsible for binding the virus to host cell receptors; therefore, it is a determining factor of pathogenicity and tropism in coronavirus. It is likely that the recombination identified in subunit 1 had an adaptive impact in BtMf-Yunnan2020, as has been suggested for other coronaviruses [121,122,123,124]. Finally, both BtMf-Yunnan2020 and MlYN20 presented an ORF7 unlike the other myotacoviruses; however, these proteins had a low reciprocal identity. Together, these differences could represent genomic adjustments of BtMf-Yunnan2020 that respond to specific interactions with *M. fimbriatus* proteins.

The landscape obtained from our small set of individuals of *M. fimbriatus* provided some indications on the dynamic of BtMf-Yunnan2020 diversity in the colony. BtMf-Yunnan2020 was a prevalent component among individuals and was the most abundant virus within the animals. The prevalence and abundance of a virus are major epidemiological factors related to host-switching [73]. A study of the prevalence and abundance over time of alphacoronaviruses and astroviruses in a colony of *M. myotis* found that peaks of viral abundance are related to colony formation and parturition periods [73]. It is possible that the epidemiological dynamics of BtMf-Yunnan2020 in *M. fimbriatus* colonies is similar to that observed in *M. myotis*, highlighting the need for seasonal studies as well as those incorporating host demographics to understand spillover risk based on changing ecophysiological characteristics in bats. We observed inter-host nucleotide diversity in the ORF1b gene of BtMf-Yunnan2020. To the best of our knowledge, there are no studies of inter-host diversity of alphacoronaviruses genomes in natural bat populations. In SARS-CoV-2, inter-host diversity has been a source of adaptation to the new host [125,126,127]. It is likely that inter-host variation, particularly in the spike gene of BtMf-Yunnan2020, plays a role in the appearance of new strains that could produce waves of infection in the colony. Another phenomenon evidenced in our data was coinfection. In individual 8, the BtMf-Yunnan2020 genome co-existed with pedacoviruses sequences. A previous study showed a significant frequency of coronavirus coinfection in bats from China. Coinfection between alphacoronaviruses of different subgenera could allow the emergence of new varieties by recombination [28]. This could be important to obtain the whole genomes of coronaviruses in natural bat populations. These studies could give an accurate assessment of the extension and implications of recombination, coinfection, and inter-individual variation in virus diversity.

#### 4.3.2. The Implications of *M. fimbriatus* Natural History on BtMf-Yunnan2020 Evolution

The hosts of myotacoviruses have a close phylogenetic and ecological history in common. This shared history probably has sculpted the evolution of the BtMf-Yunnan2020 genome. The host species of myotacoviruses belong to the genus *Myotis* and are endemic to China [37,128,129]. Phylogenetic analyses of *Myotis* genus showed that *M. fimbriatus* and *M. ricketii* are part of the same clade and are closely related, while *M. laniger* and *M. davidii* belong to a different clade [37,41]. Moreover, biogeographical analyses suggest that these bats shared similar patterns of population divergence, thus evolutionary pressures and selection may have optimized similar traits [130,131]. During the Pleistocene, the uplift of the Tibetan plate created the geographical conditions that led to the emergence of populations with different genetic diversity in *M. davidii* and *M. ricketii* [130,131]. The phylogenetic and biogeographical relation of these bats is reflected in the overlapping of their ecological niches. These four species are insectivores and *M. ricketii* also eats fish. These bats inhabit near bodies of water and roost in caves [37,41]. These species could co-roost in the same cave, as has been observed between *M. laniger* and *M. fimbriatus* or between *M. ricketti* and *M. fimbriatus* [37,41]. In this context, alphacoronavirus jumps between these bats are plausible. It is likely that the genomes of BtMf-Yunnan2020 and MlYN20 are related by an ancestral jump between *M. fimbriatus* and *M. laniger*. The inconsistency between the phylogenetic topology of myotacoviruses and hosts supports this affirmation. This event would be facilitated by co-roosting between these species, which is not uncommon in small vespertilionid species (personal observation). The co-roosting would also explain the recombination in the BtMf-Yunnan2020 spike protein involving BtMr-SAX2011 virus from *M. ricketti*. It is possible that this recombination was retained by allowing a better binding of the spike protein to the cellular receptors of *M. fimbriatus*. This scenario is based on the close phylogenetic relationship between *M. fimbriatus* and *M. ricketti*, which would have facilitated recombination in a coinfected bat. A final protagonist of this hypothesis is Yunnan. The four myotacovirus sequences were sampled in this province or nearby locations. Then, the different events that gave rise to the emergence of BtMf-Yunnan2020 possibly occurred in caves or even tree-hollows of this diversity hotspot; in either case, close physical associations between bats may facilitate the spread of pathogens.

We identified several factors that limited our study of the viral diversity of *M. fimbriatus*. The sample size was small. The twenty individuals analyzed do not represent a significant sample of the bat population in the cave. A more significant sampling could enrich the initial results of the current work. Rectal swabs have the potential to be indicators of virus replication in and shedding from bats. However, other types of sampling, for example skin swabs, could be used to characterize the total viral diversity present in a given environment, not limited to virus shedding by bats [81]. Our study enriched viral sequences using Twist’s Pan-Viral Panel. This capture system was designed to identify viral human pathogens [132]. Consequently, this strategy introduces a bias in the identification of virus diversity in bats. In the context of this strategy, it is more likely to recover viruses from families that are included in the panel and that present a certain degree of similarity to the capture sequences. This strategy can also be a confounding factor in the analysis of abundance, as it is difficult to differentiate the abundance produced by the enrichment system from the biological abundance of the virus. It is for this reason that we describe abundance as a measure of the reliability of sequences, without delving into the biological and ecological implications. At the same time, the enrichment system amplifies the viral sequences in the presence of a significant abundance of host sequences, an advantageous feature in the case of the rectal swabs used in our work [133]. Moreover, target enrichment yields full, deeply covered viral genomes from materials with Ct values, suggesting that amplicon sequencing would be likely to fail [134]. Despite the bias introduced by the target enrichment, our results demonstrate the ability of this system to capture viral diversity in mammals, sometimes recovering sequences with high divergence from available bat virus sequences. Finally, target enrichment strategies have recently emerged that seek to capture viral diversity in bats and other mammals [96,135].

## 5. Conclusions

In conclusion, a virome is a valuable tool that not only allows the identification of the diversity of viral families of epidemiological interest; it is also a means of obtaining signals on bat ecology, and enhances our understanding of the associations between bats and the viruses they host relative to elements of behavior and morphology. The virome of *M. fimbriatus* showed evidence that all of the mechanisms involved in the generation of viral zoonotic strains are present in this bat, such as recombination, coinfection, and host-switching. These observations provide a strong rationale for studying the viral diversity of bats of the *Myotis* genus with a genomic and population approach.

## Figures and Tables

**Table 1:** Viral sequences with low representation. This table presents the taxonomic classification of viral sequences with vertebrate and/or invertebrate hosts that were represented by a few contigs. The last column indicate the groups that were used to classify the densoviruses. NA: not applicable or not available.

Target	Taxonomic Classification	Name	Host	Country	Identity	No Contigs	Total Length (bp)	No Samples	Group
KF254790.1	Unclassified Orthobunyavirus	Orthobunyavirus FSL2923	*Homo sapiens*	Peru	96.63	1	141	1	NA
MG747600.1	Orthobunyavirus	Oropouche virus	*Homo sapiens*	Brazil	98.85	1	200	1	NA
KF170225.1	Unclassified Nodaviridae	Bat nodavirus	*Eptesicus serotinus*	France	87.77	1	681	1	NA
MW897047.1	Unclassified Nodaviridae	Xinjiang sediment noda-like virus 1	Environmental sample	China	86.94	1	490	1	NA
MT044485.1	Cytomegalovirus	Human betaherpesvirus 5	*Homo sapiens*	Australia	100.00	1	227	1	NA
AB850652.1	Lymphocryptovirus	Human gammaherpesvirus 4	*Homo sapiens*	China	97.73	1	109	1	NA
OM953881.1	Unclassified Genomoviridae	Flumine genomovirus 3	River water	New Zealand	100.00	1	272	1	NA
OL704847.1	Unclassified Gemykibivirus	Molossus molossus associated gemykibivirus 5	*Molossus molossus*	Argentina	100.00	1	150	1	NA
MW046351.1	Unclassified Ambidensovirus	Passer montanus ambidensovirus	*Passer montanus*	Australia	92.25	1	260	1	NA
AF036333.1	Unclassified Densovirinae	Diatraea saccharalis densovirus	*Diatraea saccharalis*	NA	99.33	1	150	1	NA
JN857356.1	Dipteran protoambidensovirus 1	Culex pipiens densovirus	*Culex pipiens*	China	100.00	1	150	1	NA
MK182384.1	Unclassified Densovirinae	Aedes albopictus densovirus 7	*Aedes albopictus*	China	98.20-100.00	7	2276	7	Group 1
EF579756.1	Dipteran brevihamaparvovirus 1	Culex pipiens pallens densovirus	*Culex pipiens pallens*	China	98.20-100.00	7	2276	7	Group 1
FJ360744.1	Dipteran brevihamaparvovirus 1	Aedes aegypti densovirus 2	*Aedes aegypti*	India	99.33-100.00	2	291	2	Group 2
MF673888.1	Unclassified Densovirinae	Mosquito densovirus	*Culex sp.*	India	99.33-100.00	2	291	2	Group 2
AY751403.1	Unclassified Brevidensovirus	Aedes aegypti Thai densovirus	*Aedes aegypti Thai*	Thailand	98.52	1	135	1	Group 3
MH188046.1	Unclassified Densovirinae	Culex densovirus	Culex sp	USA	98.51	1	135	1	Group 3

## Data Availability

The raw reads sequenced in this work are available under the Bioproject PRJNA865499 in NCBI. The sequences of viruses with vertebrate hosts described in the results and in Table S5 are also available in GenBank (https://www.ncbi.nlm.nih.gov/). All other results described in this publication are available as Supplementary Material.

## Supplementary Materials

The following supporting information can be downloaded at https://www.mdpi.com/article/10.3390/v14091899/s1, Figure S1: Pairwise identity among conserved domains; Figure S2: Pairwise identity proteins; Figure S3: Alphacoronavirus spike phylogeny; Figure S4: Sampling site in Yunnan province; Table S1: Information on collected samples; Table S2: Number of reads per sample in the main steps of bioinformatic analysis; Table S3: BLASTN results; Table S4: Number of reads per family and sample; Table S5: Information of viral sequences with vertebrate hosts; File S1: contigs sequences.

## References

[1] He B., Zhang Y., Xu L., Yang W., Yang F., Feng Y., Xia L., Zhou J., Zhen W., Feng Y. (2014). Identification of Diverse Alphacoronaviruses and Genomic Characterization of a Novel Severe Acute Respiratory Syndrome-like Coronavirus from Bats in China. J. Virol..

[2] Hu B., Zeng L.-P., Yang X.-L., Ge X.-Y., Zhang W., Li B., Xie J.-Z., Shen X.-R., Zhang Y.-Z., Wang N. (2017). Discovery of a Rich Gene Pool of Bat SARS-Related Coronaviruses Provides New Insights into the Origin of SARS Coronavirus. PLoS Pathog..

[3] Fan Y., Zhao K., Shi Z.-L., Zhou P. (2019). Bat Coronaviruses in China. Viruses.

[4] Irving A.T., Ahn M., Goh G., Anderson D.E., Wang L.-F. (2021). Lessons from the Host Defences of Bats, a Unique Viral Reservoir. Nature.

[5] Chua K.B., Koh C.L., Hooi P.S., Wee K.F., Khong J.H., Chua B.H., Chan Y.P., Lim M.E., Lam S.K. (2002). Isolation of Nipah Virus from Malaysian Island Flying-Foxes. Microbes Infect..

[6] Leroy E.M., Kumulungui B., Pourrut X., Rouquet P., Hassanin A., Yaba P., Délicat A., Paweska J.T., Gonzalez J.-P., Swanepoel R. (2005). Fruit Bats as Reservoirs of Ebola Virus. Nature.

[7] Pourrut X., Souris M., Towner J.S., Rollin P.E., Nichol S.T., Gonzalez J.-P., Leroy E. (2009). Large Serological Survey Showing Cocirculation of Ebola and Marburg Viruses in Gabonese Bat Populations, and a High Seroprevalence of Both Viruses in Rousettus Aegyptiacus. BMC Infect. Dis..

[8] Biek R., Walsh P.D., Leroy E.M., Real L.A. (2006). Recent Common Ancestry of Ebola Zaire Virus Found in a Bat Reservoir. PLoS Pathog..

[9] Olival K.J., Hosseini P.R., Zambrana-Torrelio C., Ross N., Bogich T.L., Daszak P. (2017). Host and Viral Traits Predict Zoonotic Spillover from Mammals. Nature.

[10] Brook C.E., Dobson A.P. (2015). Bats as “special” Reservoirs for Emerging Zoonotic Pathogens. Trends Microbiol..

[11] Wang L.-F., Walker P.J., Poon L.L.M. (2011). Mass Extinctions, Biodiversity and Mitochondrial Function: Are Bats “special” as Reservoirs for Emerging Viruses?. Curr. Opin. Virol..

[12] Glennon N.B., Jabado O., Lo M.K., Shaw M.L. (2015). Transcriptome Profiling of the Virus-Induced Innate Immune Response in Pteropus Vampyrus and Its Attenuation by Nipah Virus Interferon Antagonist Functions. J. Virol..

[13] Zhou P., Tachedjian M., Wynne J.W., Boyd V., Cui J., Smith I., Cowled C., Ng J.H.J., Mok L., Michalski W.P. (2016). Contraction of the Type I IFN Locus and Unusual Constitutive Expression of IFN-α in Bats. Proc. Natl. Acad. Sci. USA.

[14] Ahn M., Anderson D.E., Zhang Q., Tan C.W., Lim B.L., Luko K., Wen M., Chia W.N., Mani S., Wang L.C. (2019). Dampened NLRP3-Mediated Inflammation in Bats and Implications for a Special Viral Reservoir Host. Nat. Microbiol..

[15] Batnames|Home. https://batnames.org/.

[16] Mammal Diversity Database.

[17] Kunz T.H. (1982). Ecology of Bats.

[18] Pretorius M., Markotter W., Keith M. (2021). Assessing the Extent of Land-Use Change around Important Bat-Inhabited Caves. BMC Zool..

[19] Willoughby A.R., Phelps K.L., Olival K.J., PREDICT Consortium (2017). A Comparative Analysis of Viral Richness and Viral Sharing in Cave-Roosting Bats. Diversity.

[20] Afelt A., Frutos R., Devaux C. (2018). Bats, Coronaviruses, and Deforestation: Toward the Emergence of Novel Infectious Diseases?. Front. Microbiol..

[21] Lei F., Qu Y., Song G., Alström P., Fjeldså J. (2015). The Potential Drivers in Forming Avian Biodiversity Hotspots in the East Himalaya Mountains of Southwest China. Integr. Zool..

[22] Yang Y., Tian K., Hao J., Pei S., Yang Y. (2004). Biodiversity and Biodiversity Conservation in Yunnan, China. Biodivers. Conserv..

[23] (2013). Yunnan Biodiversity Conservation Strategy and Action Plan 2012–2030.

[24] Li Y., Ge X., Hon C.-C., Zhang H., Zhou P., Zhang Y., Wu Y., Wang L.-F., Shi Z. (2010). Prevalence and Genetic Diversity of Adeno-Associated Viruses in Bats from China. J. Gen. Virol..

[25] Li Y., Ge X., Zhang H., Zhou P., Zhu Y., Zhang Y., Yuan J., Wang L.-F., Shi Z. (2010). Host Range, Prevalence, and Genetic Diversity of Adenoviruses in Bats. J. Virol..

[26] Wang B., Yang X.-L., Li W., Zhu Y., Ge X.-Y., Zhang L.-B., Zhang Y.-Z., Bock C.-T., Shi Z.-L. (2017). Detection and Genome Characterization of Four Novel Bat Hepadnaviruses and a Hepevirus in China. Virol. J..

[27] Ge X., Li J., Peng C., Wu L., Yang X., Wu Y., Zhang Y., Shi Z. (2011). Genetic Diversity of Novel Circular SsDNA Viruses in Bats in China. J. Gen. Virol..

[28] Ge X.-Y., Wang N., Zhang W., Hu B., Li B., Zhang Y.-Z., Zhou J.-H., Luo C.-M., Yang X.-L., Wu L.-J. (2016). Coexistence of Multiple Coronaviruses in Several Bat Colonies in an Abandoned Mineshaft. Virol. Sin..

[29] Wrobel A.G., Benton D.J., Xu P., Roustan C., Martin S.R., Rosenthal P.B., Skehel J.J., Gamblin S.J. (2020). SARS-CoV-2 and Bat RaTG13 Spike Glycoprotein Structures Inform on Virus Evolution and Furin-Cleavage Effects. Nat. Struct. Mol. Biol..

[30] Li W., Shi Z., Yu M., Ren W., Smith C., Epstein J.H., Wang H., Crameri G., Hu Z., Zhang H. (2005). Bats Are Natural Reservoirs of SARS-Like Coronaviruses. Science.

[31] Ge X.-Y., Li J.-L., Yang X.-L., Chmura A.A., Zhu G., Epstein J.H., Mazet J.K., Hu B., Zhang W., Peng C. (2013). Isolation and Characterization of a Bat SARS-like Coronavirus That Uses the ACE2 Receptor. Nature.

[32] Latinne A., Hu B., Olival K.J., Zhu G., Zhang L., Li H., Chmura A.A., Field H.E., Zambrana-Torrelio C., Epstein J.H. (2020). Origin and Cross-Species Transmission of Bat Coronaviruses in China. Nat. Commun..

[33] Hu D., Zhu C., Wang Y., Ai L., Yang L., Ye F., Ding C., Chen J., He B., Zhu J. (2017). Virome Analysis for Identification of Novel Mammalian Viruses in Bats from Southeast China. Sci. Rep..

[34] Wu Z., Yang L., Ren X., He G., Zhang J., Yang J., Qian Z., Dong J., Sun L., Zhu Y. (2016). Deciphering the Bat Virome Catalog to Better Understand the Ecological Diversity of Bat Viruses and the Bat Origin of Emerging Infectious Diseases. ISME J..

[35] Wu Z., Ren X., Yang L., Hu Y., Yang J., He G., Zhang J., Dong J., Sun L., Du J. (2012). Virome Analysis for Identification of Novel Mammalian Viruses in Bat Species from Chinese Provinces. J. Virol..

[36] Han Y., Du J., Su H., Zhang J., Zhu G., Zhang S., Wu Z., Jin Q. (2019). Identification of Diverse Bat Alphacoronaviruses and Betacoronaviruses in China Provides New Insights Into the Evolution and Origin of Coronavirus-Related Diseases. Front. Microbiol..

[37] Ruedi M., Csorba G., Lin L.K., Chou C.H. (2015). Molecular Phylogeny and Morphological Revision of Myotis Bats (Chiroptera: Vespertilionidae) from Taiwan and Adjacent China. Zootaxa.

[38] Wilson D.E., Reeder D.M. (2005). Mammal Species of the World: A Taxonomic and Geographic Reference.

[39] Simmons N.B., Seymour K.L., Habersetzer J., Gunnell G.F. (2008). Primitive Early Eocene Bat from Wyoming and the Evolution of Flight and Echolocation. Nature.

[40] Myotis Fimbriatus. http://www.bio.bris.ac.uk/research/bats/China%20bats/myotisfimbriatus.htm.

[41] Chang Y., Song S., Li A., Zhang Y., Li Z., Xiao Y., Jiang T., Feng J., Lin A. (2019). The Roles of Morphological Traits, Resource Variation and Resource Partitioning Associated with the Dietary Niche Expansion in the Fish-Eating Bat Myotis Pilosus. Mol. Ecol..

[42] Han H.-J., Wen H.-L., Zhao L., Liu J.-W., Luo L.-M., Zhou C.-M., Qin X.-R., Zhu Y.-L., Liu M.-M., Qi R. (2017). Novel Coronaviruses, Astroviruses, Adenoviruses and Circoviruses in Insectivorous Bats from Northern China. Zoonoses Public Health.

[43] Chornelia A., Lu J., Hughes A.C. (2022). How to Accurately Delineate Morphologically Conserved Taxa and Diagnose Their Phenotypic Disparities: Species Delimitation in Cryptic Rhinolophidae (Chiroptera). Front. Ecol. Evol..

[44] Bolger A.M., Lohse M., Usadel B. (2014). Trimmomatic: A Flexible Trimmer for Illumina Sequence Data. Bioinformatics.

[45] Langmead B., Salzberg S.L. (2012). Fast Gapped-Read Alignment with Bowtie 2. Nat. Methods.

[46] NCBI Resource Coordinators (2018). Database Resources of the National Center for Biotechnology Information. Nucleic Acids Res..

[47] Schneider V.A., Graves-Lindsay T., Howe K., Bouk N., Chen H.-C., Kitts P.A., Murphy T.D., Pruitt K.D., Thibaud-Nissen F., Albracht D. (2016). Evaluation of GRCh38 and de Novo Haploid Genome Assemblies Demonstrates the Enduring Quality of the Reference Assembly. bioRxiv.

[48] Li D., Liu C.-M., Luo R., Sadakane K., Lam T.-W. (2015). MEGAHIT: An Ultra-Fast Single-Node Solution for Large and Complex Metagenomics Assembly via Succinct de Bruijn Graph. Bioinformatics.

[49] Li W., Godzik A. (2006). Cd-Hit: A Fast Program for Clustering and Comparing Large Sets of Protein or Nucleotide Sequences. Bioinformatics.

[50] Bushnell B. (2014). BBMap: A Fast, Accurate, Splice-Aware Aligner.

[51] Goodacre N., Aljanahi A., Nandakumar S., Mikailov M., Khan A.S. (2018). A Reference Viral Database (RVDB) To Enhance Bioinformatics Analysis of High-Throughput Sequencing for Novel Virus Detection. mSphere.

[52] Harris R.S. (2007). Improved Pairwise Alignment of Genomic DNA. Ph. D. Thesis.

[53] Katoh K., Standley D.M. (2013). MAFFT Multiple Sequence Alignment Software Version 7: Improvements in Performance and Usability. Mol. Biol. Evol..

[54] Tamura K., Stecher G., Kumar S. (2021). MEGA11: Molecular Evolutionary Genetics Analysis Version 11. Mol. Biol. Evol..

[55] Posada D. (2008). JModelTest: Phylogenetic Model Averaging. Mol. Biol. Evol..

[56] Darriba D., Taboada G.L., Doallo R., Posada D. (2011). ProtTest 3: Fast Selection of Best-Fit Models of Protein Evolution. Bioinformatics.

[57] Ronquist F., Teslenko M., van der Mark P., Ayres D.L., Darling A., Höhna S., Larget B., Liu L., Suchard M.A., Huelsenbeck J.P. (2012). MrBayes 3.2: Efficient Bayesian Phylogenetic Inference and Model Choice across a Large Model Space. Syst. Biol..

[58] R Core Team (2021). R: A Language and Environment for Statistical Computing.

[59] Campitelli E. (2021). Ggnewscale: Multiple Fill and Colour Scales in “Ggplot2.”.

[60] Yu G., Smith D.K., Zhu H., Guan Y., Lam T.T.-Y. (2017). Ggtree: An r Package for Visualization and Annotation of Phylogenetic Trees with Their Covariates and Other Associated Data. Methods Ecol. Evol..

[61] Wang L.-G., Lam T.T.-Y., Xu S., Dai Z., Zhou L., Feng T., Guo P., Dunn C.W., Jones B.R., Bradley T. (2020). Treeio: An R Package for Phylogenetic Tree Input and Output with Richly Annotated and Associated Data. Mol. Biol. Evol..

[62] Nurk S., Meleshko D., Korobeynikov A., Pevzner P.A. (2017). MetaSPAdes: A New Versatile Metagenomic Assembler. Genome Res..

[63] Capella-Gutiérrez S., Silla-Martínez J.M., Gabaldón T. (2009). TrimAl: A Tool for Automated Alignment Trimming in Large-Scale Phylogenetic Analyses. Bioinformatics.

[64] Guindon S., Dufayard J.-F., Lefort V., Anisimova M., Hordijk W., Gascuel O. (2010). New Algorithms and Methods to Estimate Maximum-Likelihood Phylogenies: Assessing the Performance of PhyML 3.0. Syst. Biol..

[65] Lin X.-D., Wang W., Hao Z.-Y., Wang Z.-X., Guo W.-P., Guan X.-Q., Wang M.-R., Wang H.-W., Zhou R.-H., Li M.-H. (2017). Extensive Diversity of Coronaviruses in Bats from China. Virology.

[66] Tan Z., Nie F.Y., Zhang Y.Z. (2021). Comparation of Mammalian Active Virome Structures and with Host-Virus Interactions in Sympatric Communities.

[67] Pensaert M.B., de Bouck P. (1978). A New Coronavirus-like Particle Associated with Diarrhea in Swine. Arch. Virol..

[68] Lazov C.M., Belsham G.J., Bøtner A., Rasmussen T.B. (2021). Full-Genome Sequences of Alphacoronaviruses and Astroviruses from Myotis and Pipistrelle Bats in Denmark. Viruses.

[69] Wohlgemuth N., Honce R., Schultz-Cherry S. (2019). Astrovirus Evolution and Emergence. Infect. Genet. Evol..

[70] Zhu H.C., Chu D.K.W., Liu W., Dong B.Q., Zhang S.Y., Zhang J.X., Li L.F., Vijaykrishna D., Smith G.J.D., Chen H.L. (2009). Detection of Diverse Astroviruses from Bats in China. J. Gen. Virol..

[71] Accession No. YP_009664789, Mamastrovirus 18 Nucleotide [Internet]. Bethesda (MD): National Library of Medicine (US), National Center for Biotechnology Information. https://www.ncbi.nlm.nih.gov/protein/YP_009664789.1/.

[72] Chu D.K.W., Poon L.L.M., Guan Y., Peiris J.S.M. (2008). Novel Astroviruses in Insectivorous Bats. J. Virol..

[73] Drexler J.F., Corman V.M., Wegner T., Tateno A.F., Zerbinati R.M., Gloza-Rausch F., Seebens A., Müller M.A., Drosten C. (2011). Amplification of Emerging Viruses in a Bat Colony. Emerg. Infect. Dis..

[74] Jansen van Vuren P., Allam M., Wiley M.R., Ismail A., Storm N., Birkhead M., Markotter W., Palacios G., Paweska J.T. (2018). A Novel Adenovirus Isolated from the Egyptian Fruit Bat in South Africa Is Closely Related to Recent Isolates from China. Sci. Rep..

[75] Ogawa H., Kajihara M., Nao N., Shigeno A., Fujikura D., Hang’ombe B.M., Mweene A.S., Mutemwa A., Squarre D., Yamada M. (2017). Characterization of a Novel Bat Adenovirus Isolated from Straw-Colored Fruit Bat (Eidolon Helvum). Viruses.

[76] Ehmann R., Brandes K., Antwerpen M., Walter M., Schlippenbach K.V., Stegmaier E., Essbauer S., Bugert J., Teifke J.P., Meyer H. (2021). Molecular and Genomic Characterization of a Novel Equine Molluscum Contagiosum-like Virus. J. Gen. Virol..

[77] Coronaviridae-Positive Sense RNA Viruses-Positive Sense RNA Viruses (2011)-ICTV. https://talk.ictvonline.org/ictv-reports/ictv_9th_report/positive-sense-rna-viruses-2011/w/posrna_viruses/222/coronaviridae.

[78] Martin D.P., Murrell B., Golden M., Khoosal A., Muhire B. (2015). RDP4: Detection and Analysis of Recombination Patterns in Virus Genomes. Virus Evol..

[79] Li L., Victoria J.G., Wang C., Jones M., Fellers G.M., Kunz T.H., Delwart E. (2010). Bat Guano Virome: Predominance of Dietary Viruses from Insects and Plants plus Novel Mammalian Viruses. J. Virol..

[80] Salmier A., Tirera S., de Thoisy B., Franc A., Darcissac E., Donato D., Bouchier C., Lacoste V., Lavergne A. (2017). Virome Analysis of Two Sympatric Bat Species (Desmodus Rotundus and Molossus Molossus) in French Guiana. PLoS ONE.

[81] Paskey A.C., Ng J.H.J., Rice G.K., Chia W.N., Philipson C.W., Foo R.J.H., Cer R.Z., Long K.A., Lueder M.R., Frey K.G. (2020). The Temporal RNA Virome Patterns of a Lesser Dawn Bat (Eonycteris Spelaea) Colony Revealed by Deep Sequencing. Virus Evol..

[82] Ge X., Li Y., Yang X., Zhang H., Zhou P., Zhang Y., Shi Z. (2012). Metagenomic Analysis of Viruses from Bat Fecal Samples Reveals Many Novel Viruses in Insectivorous Bats in China. J. Virol..

[83] He B., Li Z., Yang F., Zheng J., Feng Y., Guo H., Li Y., Wang Y., Su N., Zhang F. (2013). Virome Profiling of Bats from Myanmar by Metagenomic Analysis of Tissue Samples Reveals More Novel Mammalian Viruses. PLoS ONE.

[84] Li Y., Altan E., Reyes G., Halstead B., Deng X., Delwart E. (2021). Virome of Bat Guano from Nine Northern California Roosts. J. Virol..

[85] Su S., Wong G., Shi W., Liu J., Lai A.C.K., Zhou J., Liu W., Bi Y., Gao G.F. (2016). Epidemiology, Genetic Recombination, and Pathogenesis of Coronaviruses. Trends Microbiol..

[86] Woo P.C.Y., Lau S.K.P., Lam C.S.F., Lau C.C.Y., Tsang A.K.L., Lau J.H.N., Bai R., Teng J.L.L., Tsang C.C.C., Wang M. (2012). Discovery of Seven Novel Mammalian and Avian Coronaviruses in the Genus Deltacoronavirus Supports Bat Coronaviruses as the Gene Source of Alphacoronavirus and Betacoronavirus and Avian Coronaviruses as the Gene Source of Gammacoronavirus and Deltacoronavirus. J. Virol..

[87] van Marle G., Dobbe Jessika C., Gultyaev Alexander P., Luytjes W., Spaan Willy J.M., Snijder Eric J. (1999). Arterivirus Discontinuous MRNA Transcription Is Guided by Base Pairing between Sense and Antisense Transcription-Regulating Sequences. Proc. Natl. Acad. Sci. USA.

[88] Vetterlein W., Hesse R. (1965). Electron Microscopic Picture of Viral Hepatitis in Man and Mouse. Arch. Für Exp. Vet..

[89] Hendley J.O., Fishburne H.B., Gwaltney J.M. (1972). Coronavirus Infections in Working Adults. Am. Rev. Respir. Dis..

[90] Decaro N., Mari V., von Reitzenstein M., Lucente M.S., Cirone F., Elia G., Martella V., King V.L., Di Bello A., Varello K. (2012). A Pantropic Canine Coronavirus Genetically Related to the Prototype Isolate CB/05. Vet. Microbiol..

[91] Li C., Liu Q., Kong F., Guo D., Zhai J., Su M., Sun D. (2019). Circulation and Genetic Diversity of Feline Coronavirus Type I and II from Clinically Healthy and FIP-Suspected Cats in China. Transbound. Emerg. Dis..

[92] Doyle L.P., Hutchings L.M. (1946). A Transmissible Gastroenteritis in Pigs. J. Am. Vet. Med. Assoc..

[93] Wang L., Zhang Y. (2017). Genomic Characterization of a New PRCV Variant, United States, 2014. Transbound. Emerg. Dis..

[94] Tang X.C., Zhang J.X., Zhang S.Y., Wang P., Fan X.H., Li L.F., Li G., Dong B.Q., Liu W., Cheung C.L. (2006). Prevalence and Genetic Diversity of Coronaviruses in Bats from China. J. Virol..

[95] Huang Y.-W., Dickerman A.W., Piñeyro P., Li L., Fang L., Kiehne R., Opriessnig T., Meng X.-J. (2013). Origin, Evolution, and Genotyping of Emergent Porcine Epidemic Diarrhea Virus Strains in the United States. mBio.

[96] Aguilar Pierlé S., Zamora G., Ossa G., Gaggero A., Barriga G.P. (2022). The Myotis Chiloensis Guano Virome: Viral Nucleic Acid Enrichments for High-Resolution Virome Elucidation and Full Alphacoronavirus Genome Assembly. Viruses.

[97] De Benedictis P., Schultz-Cherry S., Burnham A., Cattoli G. (2011). Astrovirus Infections in Humans and Animals-Molecular Biology, Genetic Diversity, and Interspecies Transmissions. Infect. Genet. Evol..

[98] Fischer K., Pinho Dos Reis V., Balkema-Buschmann A. (2017). Bat Astroviruses: Towards Understanding the Transmission Dynamics of a Neglected Virus Family. Viruses.

[99] Hu B., Chmura A.A., Li J., Zhu G., Desmond J.S., Zhang Y., Zhang W., Epstein J.H., Daszak P., Shi Z. (2014). Detection of Diverse Novel Astroviruses from Small Mammals in China. J. Gen. Virol..

[100] King A.M.Q., Lefkowitz E., Adams M.J., Carstens E.B. (2012). Virus Taxonomy: Ninth Report of the International Committee on Taxonomy of Viruses.

[101] Böszörményi K.P., Podgorski I.I., Vidovszky M.Z., Sós E., Benkő M., Harrach B. (2020). Full Genome Sequence Analysis of a Novel Adenovirus from a Captive Polar Bear (Ursus Maritimus). Virus Res..

[102] Sonntag M., Mühldorfer K., Speck S., Wibbelt G., Kurth A. (2009). New Adenovirus in Bats, Germany. Emerg. Infect. Dis..

[103] Zell R., Delwart E., Gorbalenya A.E., Hovi T., King A.M.Q., Knowles N.J., Lindberg A.M., Pallansch M.A., Palmenberg A.C., Reuter G. (2017). ICTV Virus Taxonomy Profile: Picornaviridae. J. Gen. Virol..

[104] Tracy S., Chapman N.M., Drescher K.M., Kono K., Tapprich W., Domingo E. (2006). Evolution of Virulence in Picornaviruses. Quasispecies: Concept and Implications for Virology.

[105] Yu J., Li X., Ao Y., Li L., Liu N., Li J., Duan Z. (2013). Identification of a Novel Picornavirus in Healthy Piglets and Seroepidemiological Evidence of Its Presence in Humans. PLoS ONE.

[106] Krumbholz A., Dauber M., Henke A., Birch-Hirschfeld E., Knowles N.J., Stelzner A., Zell R. (2002). Sequencing of Porcine Enterovirus Groups II and III Reveals Unique Features of Both Virus Groups. J. Virol..

[107] Oberste M.S., Maher K., Pallansch M.A. (2003). Genomic Evidence That Simian Virus 2 and Six Other Simian Picornaviruses Represent a New Genus in Picornaviridae. Virology.

[108] Rivadulla E., Romalde J.L. (2020). A Comprehensive Review on Human Aichi Virus. Virol. Sin..

[109] Reuter G., Boros Á., Pankovics P. (2011). Kobuviruses–A Comprehensive Review. Rev. Med. Virol..

[110] Lu L., Van Dung N., Ivens A., Bogaardt C., O’Toole A., Bryant J.E., Carrique-Mas J., Van Cuong N., Anh P.H., Rabaa M.A. (2018). Genetic Diversity and Cross-Species Transmission of Kobuviruses in Vietnam. Virus Evol..

[111] Emerson G.L., Li Y., Frace M.A., Olsen-Rasmussen M.A., Khristova M.L., Govil D., Sammons S.A., Regnery R.L., Karem K.L., Damon I.K. (2009). The Phylogenetics and Ecology of the Orthopoxviruses Endemic to North America. PLoS ONE.

[112] Zorec T.M., Kutnjak D., Hošnjak L., Kušar B., Trčko K., Kocjan B.J., Li Y., Križmarić M., Miljković J., Ravnikar M. (2018). New Insights into the Evolutionary and Genomic Landscape of Molluscum Contagiosum Virus (MCV) Based on Nine MCV1 and Six MCV2 Complete Genome Sequences. Viruses.

[113] Vermi W., Fisogni S., Salogni L., Schärer L., Kutzner H., Sozzani S., Lonardi S., Rossini C., Calzavara-Pinton P., LeBoit P.E. (2011). Spontaneous Regression of Highly Immunogenic Molluscum Contagiosum Virus (MCV)-Induced Skin Lesions Is Associated with Plasmacytoid Dendritic Cells and IFN-DC Infiltration. J. Investig. Dermatol..

[114] Chen X., Anstey A.V., Bugert J.J. (2013). Molluscum Contagiosum Virus Infection. Lancet Infect. Dis..

[115] Fox R., Thiemann A., Everest D., Steinbach F., Dastjerdi A., Finnegan C. (2012). Molluscum Contagiosum in Two Donkeys. Vet. Rec..

[116] Bennett M., Tu S.-L., Upton C., McArtor C., Gillett A., Laird T., O’Dea M. (2017). Complete Genomic Characterisation of Two Novel Poxviruses (WKPV and EKPV) from Western and Eastern Grey Kangaroos. Virus Res..

[117] Baker K.S., Leggett R.M., Bexfield N.H., Alston M., Daly G., Todd S., Tachedjian M., Holmes C.E.G., Crameri S., Wang L.-F. (2013). Metagenomic Study of the Viruses of African Straw-Coloured Fruit Bats: Detection of a Chiropteran Poxvirus and Isolation of a Novel Adenovirus. Virology.

[118] Kocherhans R., Bridgen A., Ackermann M., Tobler K. (2001). Completion of the Porcine Epidemic Diarrhoea Coronavirus (PEDV) Genome Sequence. Virus Genes.

[119] Jantraphakorn Y., Viriyakitkosol R., Jongkaewwattana A., Kaewborisuth C. (2021). Interaction Between PEDV and Its Hosts: A Closer Look at the ORF3 Accessory Protein. Front. Vet. Sci..

[120] Bernasconi C., Guscetti F., Utiger A., Reeth K., Ackermann M., Pospischil A. (1994). Experimental Infection of Gnotobiotic Piglets With a Cell Culture Adapted Porcine Epidemic Diarrhoea Virus: Clinical, Histopathological and Immunohistochemical Findings. Immunobiology of Viral Infections, Proceedings of the 3rd Congress of the European Society for Veterinary Virology, Interlaken, Switzerland, 4–7 September 1994.

[121] Peng G., Sun D., Rajashankar K.R., Qian Z., Holmes K.V., Li F. (2011). Crystal Structure of Mouse Coronavirus Receptor-Binding Domain Complexed with Its Murine Receptor. Proc. Natl. Acad. Sci. USA.

[122] Sánchez C.M., Izeta A., Sánchez-Morgado J.M., Alonso S., Sola I., Balasch M., Plana-Durán J., Enjuanes L. (1999). Targeted Recombination Demonstrates That the Spike Gene of Transmissible Gastroenteritis Coronavirus Is a Determinant of Its Enteric Tropism and Virulence. J. Virol..

[123] Kuo L., Godeke G.-J., Raamsman M.J.B., Masters P.S., Rottier P.J.M. (2000). Retargeting of Coronavirus by Substitution of the Spike Glycoprotein Ectodomain: Crossing the Host Cell Species Barrier. J. Virol..

[124] Menachery V.D., Yount B.L., Debbink K., Agnihothram S., Gralinski L.E., Plante J.A., Graham R.L., Scobey T., Ge X.-Y., Donaldson E.F. (2015). A SARS-like Cluster of Circulating Bat Coronaviruses Shows Potential for Human Emergence. Nat. Med..

[125] Rochman N.D., Wolf Y.I., Faure G., Mutz P., Zhang F., Koonin E.V. (2021). Ongoing Global and Regional Adaptive Evolution of SARS-CoV-2. Proc. Natl. Acad. Sci. USA.

[126] Kistler K.E., Huddleston J., Bedford T. (2022). Rapid and Parallel Adaptive Mutations in Spike S1 Drive Clade Success in SARS-CoV-2. Cell Host Microbe.

[127] Mendiola-Pastrana I.R., López-Ortiz E., de la Loza-Zamora J.G.R., González J., Gómez-García A., López-Ortiz G. (2022). SARS-CoV-2 Variants and Clinical Outcomes: A Systematic Review. Life.

[128] Wang S.-Q., Li Y.-J., Yin A.-G., Zhang W., Jiang J.-J., Wang W.-L., Hu M. (2016). The Complete Mitochondrial Genome of David’s Myotis, Myotis Davidii (Myotis, Vespertilionidae). Mitochondrial DNA Part A.

[129] Hao X. (2019). Complete Mitochondrial Genome of the East Asian Fish-Eating Bat: Myotis Ricketti (Chiroptera, Vespertilionidae). Mitochondrial DNA Part B.

[130] You Y., Sun K., Xu L., Wang L., Jiang T., Liu S., Lu G., Berquist S.W., Feng J. (2010). Pleistocene Glacial Cycle Effects on the Phylogeography of the Chinese Endemic Bat Species, Myotis Davidii. BMC Evol. Biol..

[131] Lu G., Lin A., Luo J., Blondel D.V., Meiklejohn K.A., Sun K., Feng J. (2013). Phylogeography of the Rickett’s Big-Footed Bat, Myotis Pilosus(Chiroptera: Vespertilionidae): A Novel Pattern of Genetic Structure of Bats in China. BMC Evol. Biol..

[132] Twist Pan-Viral Panel|Twist Bioscience. https://www.twistbioscience.com/resources/protocol/twist-pan-viral-panel.

[133] Gaudin M., Desnues C. (2018). Hybrid Capture-Based Next Generation Sequencing and Its Application to Human Infectious Diseases. Front. Microbiol..

[134] Paskey A.C., Frey K.G., Schroth G., Gross S., Hamilton T., Bishop-Lilly K.A. (2019). Enrichment Post-Library Preparation Enhances the Sensitivity of High-Throughput Sequencing-Based Detection and Characterization of Viruses from Complex Samples. BMC Genom..

[135] Comprehensive Viral Research Panel-Twist Bioscience. https://www.twistbioscience.com/products/ngs/fixed-panels/comprehensive-viral-research-panel?sbrc=1ZlXZV5FMyQc3dE81mG82bA%3D%3D%24Yyrt_UOJqXKCkEp6eVX9KQ%3D%3D.

