# Supplementary material for: Myotis fimbriatus Virome, a Window to Virus Diversity and Evolution in the Genus Myotis
Source: Viruses. 2022 Aug 27;14(9):1899. doi: 10.3390/v14091899 (PMC9505981; doi:10.3390/v14091899)
Supplement: Supplementary file 1 [file viruses-14-01899-s001.zip › Supplementary/SupplementaryFigures.pdf]

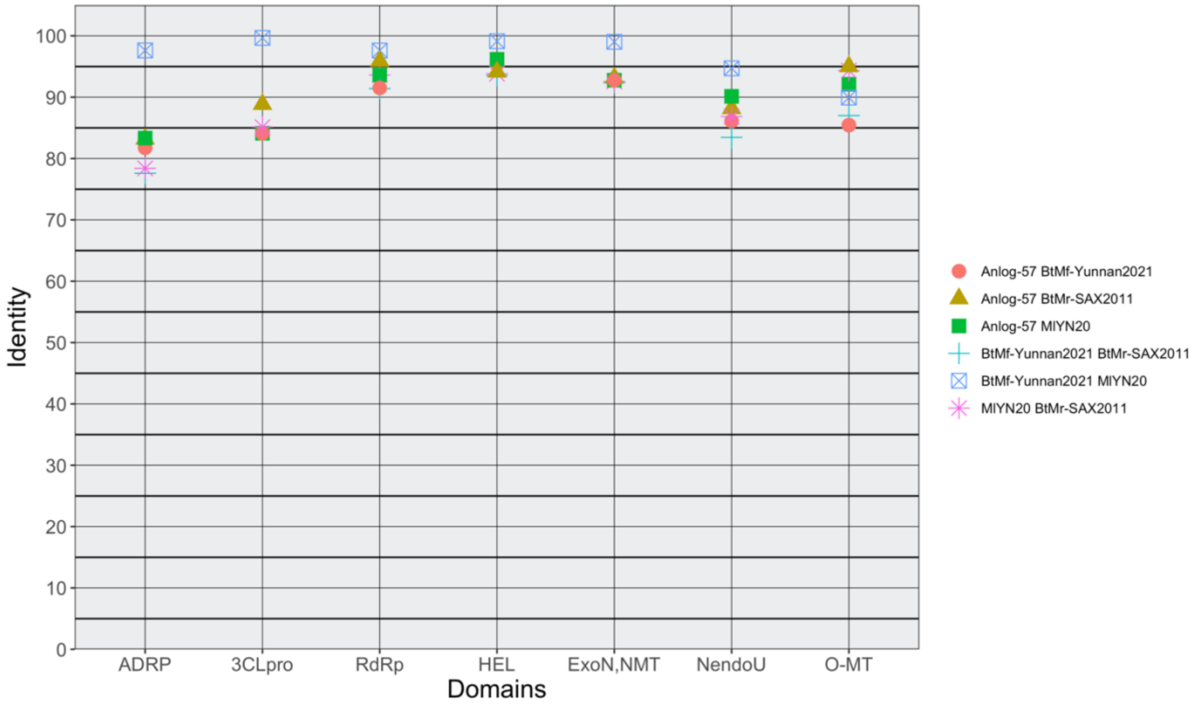

Figure S1. Pairwise identity among conserved domains. Amino acid identity was estimated between the conserved domains in myotacovirus sequences. ADRP: ADP-ribose-1'-phosphatase, 3CLpro: 3C-like protease, RdRp: RNA dependent RNA polymerase, HEL: helicase, ExoN: 3'-5' exoribonuclease, NMT: N7-methyltransferase, NendoU: nidoviral uridylate-specific endoribonuclease, O-MT: ribose-2′-O-methyltransferase.

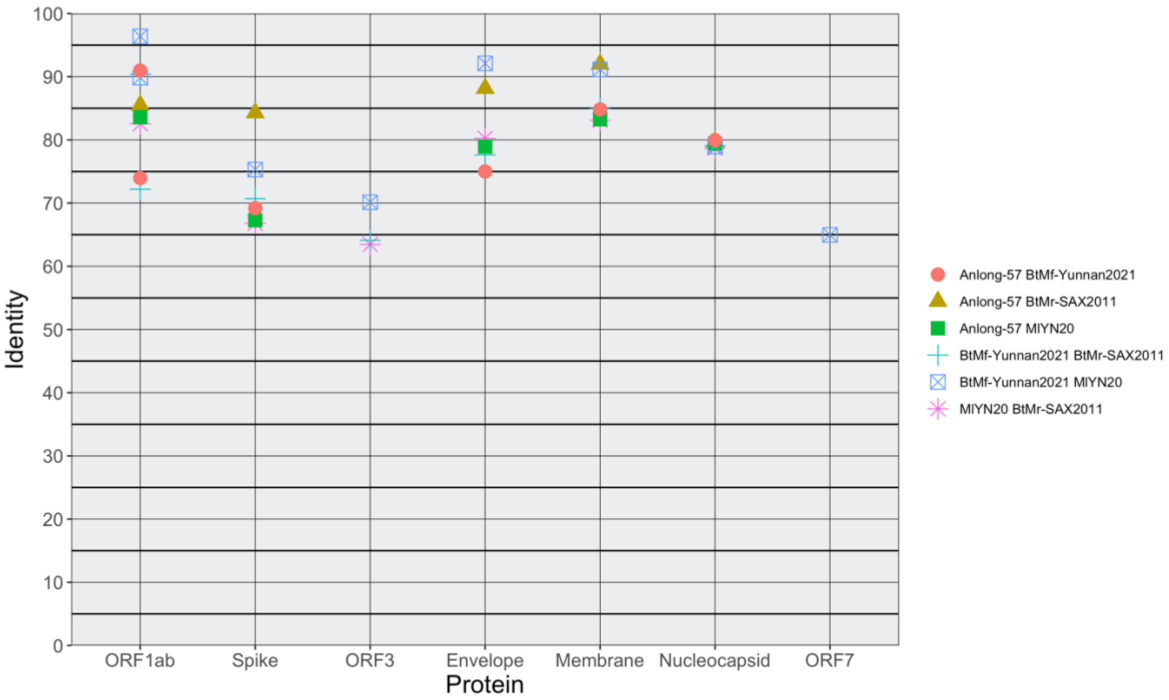

Figure S2. Pairwise identity proteins. Amino acid identity among myotacovirus proteins.

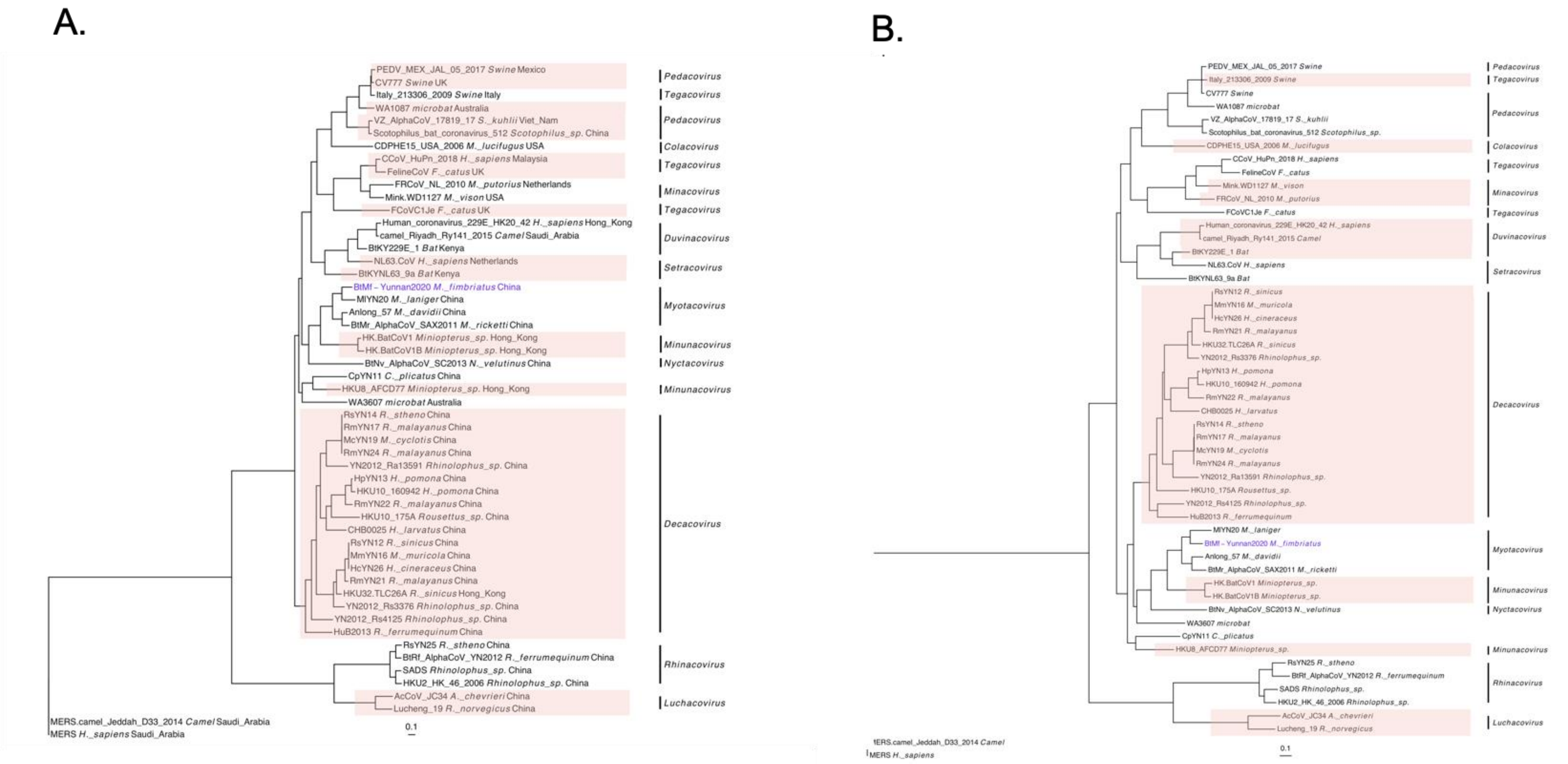

Figure S3. Alphacoronavirus spike phylogeny. (A) Phylogenetic tree of the alphacoronavirus spike protein sequences (1,268 a.a). The tree inference was based on the LG+I+G model. (B) Phylogenetic tree of spike subunit 1 (469 a.a). The inference was made under the Whelan and Goldman (WAG) model with gamma distributed rate variation among sites and proportion of invariable sites.

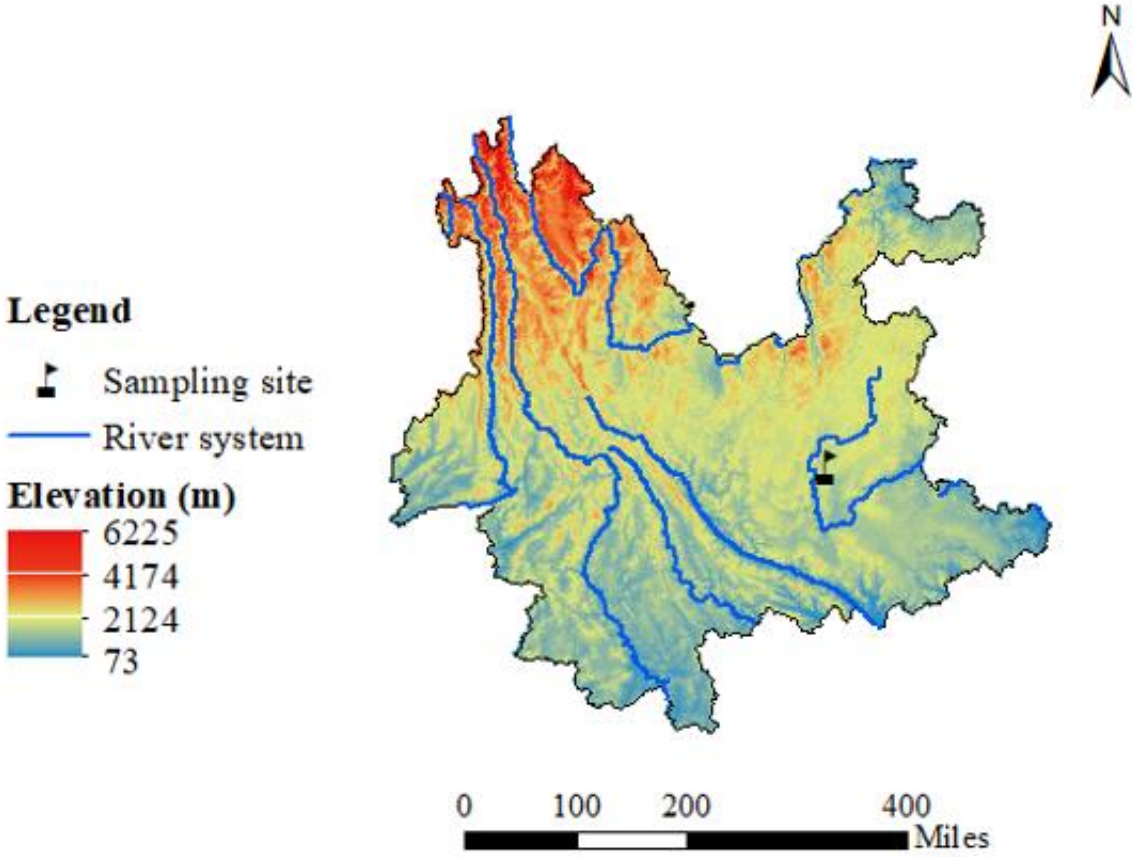

Figure S4. Sampling site in Yunnan province. This map represents the relief and some of the main rivers of the Yunnan province in China. The cave is located close to the Nanpan river in Boluohei county.
