# Supplementary material for: Myotis fimbriatus Virome, a Window to Virus Diversity and Evolution in the Genus Myotis
Source: Viruses. 2022 Aug 27;14(9):1899. doi: 10.3390/v14091899 (PMC9505981; doi:10.3390/v14091899)
Supplement: Supplementary file 1 [file viruses-14-01899-s001.zip › Supplementary/TableS1.pdf]

Table S1. Information on collected samples

Individual no.	Sample type	Sampling location	Location type	Concentration of RNA [a]
1	Rectal swab	Shilin county, Kunming, Yunnan	Cave	10.5
2	Rectal swab	Shilin county, Kunming, Yunnan	Cave	4.6
3	Rectal swab	Shilin county, Kunming, Yunnan	Cave	—
4	Rectal swab	Shilin county, Kunming, Yunnan	Cave	—
5	Rectal swab	Shilin county, Kunming, Yunnan	Cave	—
6	Rectal swab	Shilin county, Kunming, Yunnan	Cave	—
7	Rectal swab	Shilin county, Kunming, Yunnan	Cave	5.38
8	Rectal swab	Shilin county, Kunming, Yunnan	Cave	—
9	Rectal swab	Shilin county, Kunming, Yunnan	Cave	—
10	Rectal swab	Shilin county, Kunming, Yunnan	Cave	—
11	Rectal swab	Shilin county, Kunming, Yunnan	Cave	4.4
12	Rectal swab	Shilin county, Kunming, Yunnan	Cave	—
13	Rectal swab	Shilin county, Kunming, Yunnan	Cave	—
14	Rectal swab	Shilin county, Kunming, Yunnan	Cave	—
15	Rectal swab	Shilin county, Kunming, Yunnan	Cave	—
16	Rectal swab	Shilin county, Kunming, Yunnan	Cave	—
17	Rectal swab	Shilin county, Kunming, Yunnan	Cave	—
18	Rectal swab	Shilin county, Kunming, Yunnan	Cave	—
19	Rectal swab	Shilin county, Kunming, Yunnan	Cave	—
20	Rectal swab	Shilin county, Kunming, Yunnan	Cave	4.8

a—, the concentration was below 5 ng/μL and tested by Qubit.
